# Preschoolers rely on rich speech representations to process variable speech

**DOI:** 10.1111/cdev.13922

**Published:** 2023-04-10

**Authors:** Margaret Cychosz, Tristan Mahr, Benjamin Munson, Rochelle Newman, Jan R. Edwards

**Affiliations:** 1 Department of Linguistics, University of California, Los Angeles, Los Angeles, California, USA; 2 Waisman Center, University of Wisconsin– Madison, Madison, Wisconsin, USA; 3 Department of Speech- Language- Hearing Sciences, University of Minnesota, Twin Cities, Minneapolis, Minnesota, USA; 4 Department of Hearing and Speech Sciences and Maryland Language Science Center, University of Maryland, College Park, College Park, Maryland, USA

## Abstract

To learn language, children must map variable input to categories such as phones and words. How do children process variation and distinguish between variable pronunciations (“shoup” for *soup*) versus new words? The unique sensory experience of children with cochlear implants, who learn speech through their device’s degraded signal, lends new insight into this question. In a mispronunciation sensitivity eyetracking task, children with implants (*N* = 33), and typical hearing (*N* = 24; 36–66 months; 36F, 19M; all non- Hispanic white), with larger vocabularies processed known words faster. But children with implants were less sensitive to mispronunciations than typical hearing controls. Thus, children of all hearing experiences use lexical knowledge to process familiar words but require detailed speech representations to process variable speech in real time.

To develop speech and learn words, young children must learn to parse words from a highly variable speech stream spoken around them. This is a daunting task as speech is sensitive to context, so word and phoneme exemplars differ from one production and speaker to the next. In absolute acoustic terms, this contextual variation—stemming from speaker pitch, rate, or accent—can be nearly as large as variation intended to be contrastive within a language. As a result, young children must disassociate *variable* word productions from *novel* word productions, in order to map truly novel words onto new referents in the environment and build a vocabulary.

Adult listeners process this speech variation easily, efficiently factoring in contextual factors in speech stemming from speaking rate, sentential context, and speaker identity or accent (Bradlow & Bent, 2008; Newman & Sawusch, 2009; Reinisch, 2016; Steffman, 2019). For example, an adult listener will consider their interlocutor’s speaking rate to correctly classify temporally based contrasts such as /k–g/ with differing voice onset times (Maslowski et al., 2019). In this case, the listener may observe that the *absolute* voice onset time of [k] varies, but they can nevertheless classify the phoneme appropriately by computing the interlocutor’s speech rate. And while even adult listeners initially struggle to process some forms of variation, such as accented speech (Bent et al., 2016), it is widely known that adults efficiently process many forms of systematic variation in speech. This ability allows them to, for example, quickly differentiate between variable versus novel word productions and determine if a novel pronunciation warrants a new entry in the lexicon (Marslen-Wilson, 1987; Marslen-Wilson & Welsh, 1978).

How do young children learn to cope with rampant speech variation that they hear in their input? When does speech variation indicate a novel accent, or a different gender, versus a new word to be learned? Without the ability to adjust for variation, children would inappropriately classify every contextual variant (e.g., [t^h^oı] or [ɾoı] for “toy”) as a novel lexical item. Thus, some degree of insensitivity is beneficial for children’s speech processing. But children must likewise determine when a pronunciation variant has crossed the threshold of lexicality and a new referent must be mapped. In developmental research, these ideas have frequently been studied using “mispronunciation” sensitivity tasks, which assess how infants and toddlers process familiar words with slight (“dog” > “tog” [tɔg]) or more extreme ([sɔg]) pronunciation variants.

Over two decades of research using this mispronunciation sensitivity paradigm have demonstrated that infants and toddlers show sensitivity to detail in speech. For instance, classic studies found that 18- to 23-month-olds looked less at a picture of a baby upon hearing “vaby” ([veıbi]) than “baby,” but still preferred the image of the baby more than a competing image of a dog (Swingley & Aslin, 2000; see also Bailey & Plunkett, 2002; Ballem & Plunkett, 2005; Swingley & Aslin, 2002). Additional work has shown that children have graded sensitivity to speech variation: 19-month-olds looked progressively less at the image of a ball when presented with progressively greater phonological mismatches (e.g., [gɔl] > [kɔl] > [sɔl] for “ball”; White & Morgan, 2008; cf. Bailey & Plunkett, 2002; Swingley & Aslin, 2002). Similar work has found that infants in this age range (15–24 months) are sensitive to vowel mispronunciations (Mani & Plunkett, 2007). And sensitivity to mispronunciations continues to be observed into the preschool years (e.g., 3–6 years, Creel, 2012), including for lexical tone (Wewalaarachchi & Singh, 2020), with children becoming more sensitive to mispronunciations as they age (between 2;6 and 4;10 [years;months], Law & Edwards, 2015). (See Von Holzen and Bergmann (2021) for a recent meta-analysis and Pomper et al. (2019) for comparisons of toddlers with and without diagnoses of autism spectrum disorder.)

Thus, from infancy, children are sensitive to both vocalic and consonantal mispronunciations and show graded awareness of these mispronunciations into toddlerhood. Mispronunciation sensitivity—the ability to detect, not disregard, sublexical variation—then appears to improve as toddlers age: children become progressively less likely to associate mispronounced words like “shoup” with the corresponding image of soup. This sensitivity to mispronunciations is critical for speech and language development: if a child cannot detect the phonetic differences between words, such as “buck” and “puck,” that child would be less likely to map buck to a novel object in the environment, inhibiting vocabulary growth. Thus, mispronunciation sensitivity indicates the maturity of children’s phonological representations which, in turn, dictates how they interpret speech variation in their environments.

The outstanding question is *how* children develop this perceptual flexibility that both permits robust processing of speech variation and makes room for novel word learning. Individual difference analyses have shown that children’s online word recognition of *correct* pronunciations (i.e., real words) improves with age (Fernald et al., 2006). Studies have also demonstrated that children with larger vocabularies, who hear more speech directed to them from caregivers in their homes, process these correctly pronounced words faster (aged 1;6–4;4: Hurtado et al., 2008; Mahr & Edwards, 2018; Weisleder & Fernald, 2013). Yet, there does not appear to be a reliable effect of vocabulary size upon mispronunciation sensitivity, at least before 2;0 (Bailey & Plunkett, 2002; Swingley & Aslin, 2000) with a recent meta-analysis not finding reliable effects of age or receptive vocabulary on the outcome between 0;6 and 2;0 (though the authors caution that the null effect of vocabulary could be due to a dearth of studies that include the measure; Von Holzen & Bergmann, 2021). It is only between 2;0 and 3;10 that a facilitative effect of vocabulary size for mispronunciation sensitivity has been shown—children with larger vocabularies are less likely to attribute mispronunciations to familiar items—suggesting that age and vocabulary effects only emerge later in toddlerhood (Law & Edwards, 2015; Swingley, 2016). This developmental pattern is potentially due to a restructuring of the lexicon and refinement of phonological representations with age. Indeed, vocabulary size is a stronger predictor of performance on mispronunciation tasks than age alone during this later developmental stage (Law & Edwards, 2015; but see Pomper et al., 2019 who did not replicate the vocabulary effect in 2- to 3-year-olds with autism spectrum disorder diagnoses).

## The speech-language experience of children with cochlear implants

In the current study, we assess how children process speech variation by extending the mispronunciation sensitivity paradigm to a population with vastly different sensory and speech-language experiences: children with cochlear implants (CIs). A CI is a prosthetic device that bypasses the middle ear to directly stimulate the cochlea and partially restore the sensation of hearing for individuals with severe to profound hearing loss. The children in this study received their CI(s) between the ages of 0;6 and 3;9. Prior to implantation, they had little to no exposure to oral language or speech input. Thus, they did not develop a vocabulary at the same pace as their peers with typical hearing (TH) because the children with CIs came from aural/oral households and did not acquire a sign language vocabulary. Nor did the children with CIs experience spoken speech variation, stemming either from variation in their own vocal productions or variation in their caregiver’s speech input (Fagan, 2014; Houston et al., 2012). (Some children who receive CIs are exposed to American Sign Language or varieties of home sign. Consequently, these children have been constructing a receptive vocabulary and have been exposed to motoric production variability [in the signed modality], Davidson et al., 2014.)

Postimplantation, the children with CIs continued to have a different sensory experience than their TH peers. Because the CI stimulates the cochlea at discrete points, it breaks a continuous spectral-temporal signal (the speech envelope) into discrete components (channels), compromising the fine-grained nature of speech (for further detail see Winn & Litovsky, 2015). We refer to this hearing experience of the children with CIs as *electric hearing* to contrast it with the *acoustic hearing* that the children with TH experience.

Electric hearing—the signal that the CI conveys—affects young children’s phonological development. When the rich, continuous speech envelope is discretized into a limited number of channels, the information available to a speech learner is limited. Consequently, the phonological representations constructed on the basis of this electric input will differ from those constructed from acoustic input. Besides discretization, electric hearing also degrades the speech signal as electrodes on the CI array often interact, stimulating multiple sites along the cochlea and compromising spectral cues that are important for vowels and place of articulation contrasts (Fu & Nogaki, 2005). Goodness of implant fit (i.e., insertion depth) likewise affects the signal available to the CI user because it can systematically shape the range of frequencies and frequency-to-electrode mapping available in the signal (Fu & Shannon, 1999).

The results of learning speech from this electric input are the well-known differences in phonological processing and representations between children with CIs and TH. Children with CIs recognize fewer words during standardized tests of speech perception (Schorr et al., 2008), have poorer phonological awareness (Ambrose et al., 2012), and have less-developed phonological sensitivity (Nittrouer et al., 2016) than children with TH. These differences often persist even when the children are appropriately matched to TH controls by years of hearing experience or a measure of language skill (James et al., 2009) and are present even among adolescent CI users (Nittrouer et al., 2021). It is phonological differences, as opposed to language, which are purported to underlie a large number of differences in speech, language, and literacy attainment between children with CIs and TH (Nittrouer, 2010).

The differences in phonological outcomes among children with CIs and TH undoubtedly stem both from the time spent without oral speech models preimplantation as well as the degraded speech signal perceived post-implantation. Even early implantation does not erase these differences. Nevertheless, despite electric hearing’s characteristic degraded signal, children who receive CIs learn to process and produce speech, hitting many speech development milestones on a similar, although protracted, timescale as their TH peers (Bruggeman et al., 2021; Fagan, 2015; Tang et al., 2019). The implant’s signal itself, however, does not improve with age or cognitive development.

Thus, children with CIs in the current study have a unique sensory experience stemming from (1) the lack of oral language exposure preimplantation and (2) the compromised speech signal postimplantation. Both sensory experiences likely shape how these children process speech and language. However, the current work focuses on the second sensory experience—the compromised speech signal—to understand how a lack of access to fine, phonetic detail shapes how children process speech variation. Here we do not claim that children with CIs cannot detect variation in the speech stream. Indeed, many children who learn speech and language through CIs show remarkable auditory plasticity and acquire age-appropriate speech processing and phonological skills on par with their peers with TH (Morini et al., 2017). Instead, this work examines how *much* speech detail children with CIs are sensitive to during speech processing. Must children have access to fine-grained detail in their speech representations in order to process variable speech?

We examine this question by comparing how children with CIs and TH process correct and mispronounced words during a mispronunciation task. If both groups of children process these words similarly, it would suggest that children can compensate for degraded speech signals and learn to process variable speech efficiently. Perhaps, the children with CIs compensate via larger vocabulary growth or top-down cues from the lexicon—parameters that future work could experimentally manipulate and examine in detail. If, on the other hand, children with CIs and TH process correctly pronounced words similarly, but mispronounced words *differently*, it would suggest that robust speech representations are a prerequisite for processing variable speech. Crucially, we match the children with CIs and TH by vocabulary size and oral language exposure. Consequently, should the groups process the mispronounced words differently, we can more confidently conclude that even relatively large vocabularies cannot help children overcome the degraded speech signal that they learned from. Overall, however, the goal of this work is to examine how much detail children with CIs are sensitive to during these online speech-processing tasks.

## The current study

We carried out a variant of the mispronunciation sensitivity task (Law & Edwards, 2015; Pomper et al., 2019; Swingley & Aslin, 2000; White & Morgan, 2008), where children were presented with two photos, one familiar and one unfamiliar, and heard a correct (*soup*), mispronounced (“shoup” [ʃup]), or novel word (“cheem” [ʧim]). Children’s eye movements to the familiar object were then tracked. The children with CIs were matched to peers with TH by language skill (vocabulary size) and lifetime oral language exposure (years of hearing experience). Matching in this way allows us to control for the children’s auditory deprivation preimplantation, as well as lexical knowledge, and isolate how the degraded, electric hearing signal post-implantation impacts the ability to process variable speech in the language used around them.

We predicted that children with CIs would distinguish less reliably between correctly pronounced (*soup*) and mispronounced words (“shoup”) than TH controls owing to the degraded speech signal generated by electric hearing (i.e., children with CIs would look more to an image of soup upon hearing “shoup”). More specifically, since the children with CIs were matched to TH controls for vocabulary, we predicted that they would process correct pronunciations (*soup*) at a similar timescale as TH controls, and that group differences would instead stem directly from the children with CIs’ insensitivity to the mispronunciations (“shoup”). Together, these results would suggest that sensitivity to phonological variation during online processing in the preschool years depends on access to fine, phonetic detail, and well-specific phonological representations. In this way, our use of the mispronunciation paradigm differs from that of some previous works that used the paradigm to examine the status of children’s early linguistic representations (Bailey & Plunkett, 2002; Swingley & Aslin, 2000). Here, the fact that many children with CIs have underspecified phonological representations is assumed from previous research (Ambrose et al., 2012; James et al., 2009; Nittrouer et al., 2021). So, the current work instead employs the mispronunciation paradigm to understand how the children process variable speech by comparing their performance on the mispronunciation task to children with TH.

Finally, previous research suggests that vocabulary facilitates the online processing of both correctly produced and mispronounced words for children with TH within this age range (Law & Edwards, 2015). Consequently, we anticipate positive correlations between the magnitude of children’s sensitivity to mispronunciations and vocabulary size for both children with TH and CIs. However, categorical speech perception is likewise linked to phonetic skills in children of this age range (Rvachew, 1994; Shiller et al., 2010). The current study predicts that group differences in mispronunciation sensitivity may stem primarily from phonetic effects, not lexical (since the groups are matched for vocabulary); we additionally anticipate positive correlations between performance on the mispronunciation task and a standardized measure of the children’s phonetic skill (articulatory acumen).

## METHODS

### Participants

Data in this study came from *N* = 37 observations of children with CIs and *N* = 37 children with TH. Data from an additional 4 children with CIs were collected but were excluded due to missing eye gaze data (see Data Cleaning section). All children were non-Hispanic white, monolingual English speakers, and were participating in a larger longitudinal research program in the upper Midwest of the United States, where children’s vocabulary and phonological development were assessed annually at ages 3, 4, and 5 between 2013 and 2016.

**FIGURE 1:**
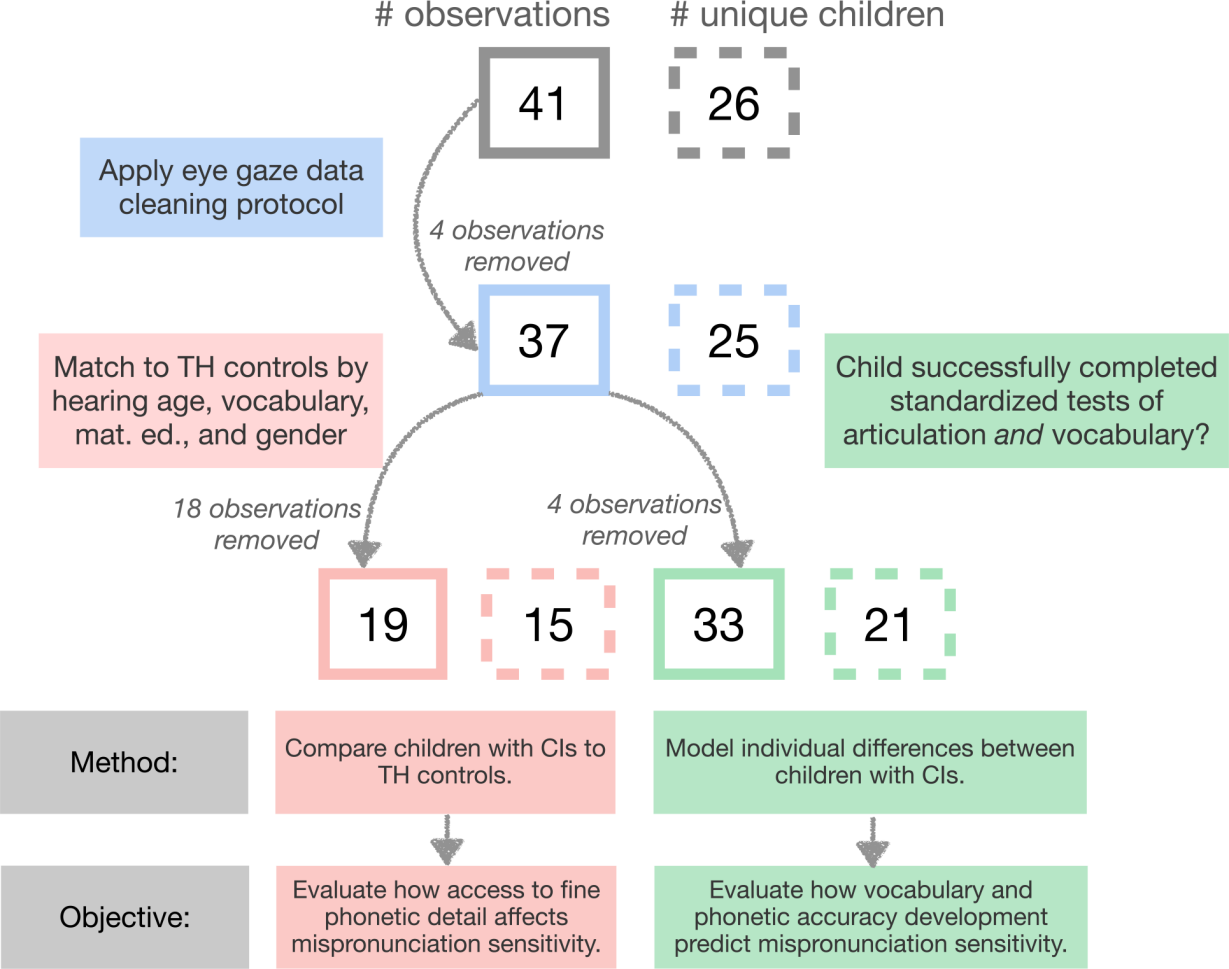
Flowchart to illustrate the effects of data cleaning and matching upon a number of children with cochlear implants examined at each analysis stage. Boxes with solid lines correspond to a number of unique observations and boxes with dotted lines to a number of unique children since observations include some children who were observed twice, 1 year apart.

The children with CIs were matched to the children with TH for hearing age, vocabulary size, gender, and maternal education using the R package ‘Matching’ (Sekhon, 2011; see Table 1 for further detail). After controlling for all relevant variables, we were able to make *N* = 19 matches. We report separate analyses for both the matched pairs (*N* = 19) and all observations of children with CIs, including those that were not matched (*N* = 37). See Figure 1 for illustration.

Maternal education was reported by the child’s caregiver(s) and was divided into seven levels for matching: (1) <high school degree, (2) equivalent of high school degree (GED), (3) high school degree, (4) technical associate’s degree, (5) some college, (6) college degree, and (7) graduate degree. To facilitate matching, while still ensuring a sufficient sample size, *N* = 9 children (*N* = 4 with CIs, and *N* = 5 with TH) contributed data from 2 out of the 3 timepoints that they were observed (e.g., at ages 3 and 4). For clarity, we refer to these repeated observations as *unique* children throughout. We explain the statistical modeling of these repeated observations in the results.

All the children with TH had typical speech and language development, per parental report. The children with TH additionally all passed a standard hearing test in at least one ear at 25 dB at 1000, 2000, and 4000 Hz. The children with CIs had severe to profound deafness in both ears. *N* = 21 had bilateral CIs, *N* = 3 had 1 hearing aid and 1 CI, and *N* = 1 child had 1 CI. The average age of CI activation was 18 months (SD = 10.6; range = 6–45). (These statistics refer to all 37 unique children with CIs who were analyzed in the current study, not just the 19 unique children with CIs who were age- and language-matched.) The children with CIs completed the Ling6 sound check prior to experimental testing to ensure CI functioning (Ling, 1976). For this test, the experimenter produces six phonemes differing in frequency (/ɑ, u, i, s, ʃ, m/), one at a time, with a cover over their mouth and lips. Children with CIs pass the check if they can repeat each sound correctly. Additional by- child audiological information is included in the Appendix.

### Stimuli

Lexical stimuli were *N* = 6 easily picturable, one-syllable CVC nouns, familiar to 90% of 30-month-olds according to MacArthur-Bates Communicative Development norms (Fenson et al., 2007). The corresponding mispronunciations were created by manipulating one feature of the initial consonant of each word (e.g., *soup* > [ʃup]). These particular consonant contrasts were chosen for audibility (e.g., /s-ʃ/ is louder than /f-ɵ/) as well as to vary consonant manner (fricative, stop, glide) across items. The *N* = 6 novel words were also CVC and were phonotactically matched to the real words on the basis of transitional probabilities between each C-V and V-C using the Hoosier Mental Lexicon (Pisoni et al., 1985; see Table 2). We do not model looking patterns in response to the novel words for the remainder of this work but report those results in the Supporting Information S1.

Visual stimuli consisted of pairs of color photos: one familiar item (e.g., soup) and one item unfamiliar to these children. The pairings remained consistent throughout the study; for example, images of soup (familiar) and bamboo steamers (unfamiliar) always appeared together. The left–right position of familiar versus unfamiliar photos on the screen was counterbalanced between trials. To maintain children’s attention, two different photos of each item were used on different trials. For the novel word trials, children were likewise presented with one familiar (unrelated to the six target familiar items) and one unfamiliar item. These items were likewise familiar to at least 90% of 30-month-olds. Photos were matched within and between trials for size, animacy, and attractiveness, per the authors’ judgment. Photos were normed by *N* = 30 children from two preschools; see Supporting Information S2 for details.

Auditory stimuli consisted of recordings of each lexical item made by a female adult in a child- directed speech register spoken in the local dialect. Lexical items were embedded in carrier sentences such as “Find the *X*!” and “See the *Y*!” To mitigate any effect of coarticulatory cues in these carrier sentences, neutral sentences with lexical items beginning with a glottal stop, to facilitate cross-splicing, were also recorded (e.g., VC in “Find the *egg*!”). Then, the target lexical item was appended to the neutral carrier phrase (“Find the_[neutral]_ soup_[target]_”) with 80 ms between the carrier phrase and the target item.

The duration was normalized between familiar items and their corresponding mispronunciations and novel words (e.g., familiar *soup* > mispronunciation [ʃup] > novel word [ʧim]). The intensity was normalized between all items.

### Task procedure

A looking-while-listening procedure was conducted (Fernald et al., 2008). Children were seated approximately 60 cm in front of a monitor screen and guided by an experimenter in the room through the task. Eye gaze was recorded using a Tobii T60XL eyetracker (60 Hz sampling rate, though this was downsampled to 50 ms windows for a rate of 20 Hz to smooth over data from adjacent frames). For each trial, photos of a familiar and unfamiliar object were centered side-by-side on a gray background. Auditory stimuli, presented at approximately 65 dB, were played from a speaker under the monitor.

Trials fell into one of three different conditions. For Correct Pronunciation trials, photos of the target familiar object and an unfamiliar object were presented with recordings of the correct pronunciation of the familiar object (e.g., *soup*).

Mispronunciation trials were identical except that the auditory stimuli were one- feature mispronunciations of the target familiar object. Novel Word trials were presented with different pairs of familiar–unfamiliar photos and the accompanying recording of the novel word. Trials were interspersed with 500 ms of a blank screen. Before beginning the experiment, the eyetracker was 5-point calibrated to each child. After approximately every 6 trials, an attention-getter was played and the experimenter ensured that the child was still sitting sufficiently close to the tracker and that their activity was being tracked.

Each child completed two experimental blocks. Each experimental block contained 12 trials per condition (Correct Pronunciation, Mispronunciation, and Novel Word) for 36 trials per block. Trials were pseudo-randomized such that (1) each block began with a Correct Pronunciation trial, (2) no more than two trials of a given condition were presented in a sequence, and (3) the correct pronunciation was never presented with its accompanying mispronunciation in the same block of trials. Children completed a standardized screening between blocks.

Each trial unfolded as follows: both photos were presented in silence for 2000 ms, to familiarize the child. Then, the presentation of the auditory stimulus was gaze-contingent: the experimental software attempted to continuously track the child’s eye gaze movements. After 10 s, if the child’s eye gaze movements had not been continuously tracked for 300 ms, the trial continued. After eye gaze verification, the carrier phrase and target word were played. This procedure attempted to guarantee that the child was looking at the screen when the audio stimulus played. For this reason, as well as some inherent durational properties of the different consonants (e.g., stops vs. fricatives), the trial duration could vary over the course of the experiment. Then, 1000 ms after the target offset, a reinforcer phrase such as *You’re doing great!* played and the images remained on the screen for another 1000 ms. Additionally, reinforcer images, and phrases were presented every 6–8 trials to maintain children’s interest. The reinforcer phrase was dropped for the observations made at age 5 to accommodate the older children.

### Data cleaning

Before data cleaning, four experimental blocks (144 trials; all from children with TH) were removed because the reinforcer phrase occurred too quickly after the auditory prompt. Next, we performed “deblinking” to account for gaze patterns that were lost due to blinking and not, for example, looking off-screen. Short windows of data (up to 150 ms) were interpolated if the child fixated on the same image before and after a missing data window. At the trial level, data quality was examined in the 250–1800 ms window following target word onset: at least 50% of the data within the window had to be valid (onscreen) to include the trial (CIs: *N* = 659/2772 trials removed; TH: *N* = 383/2952). Next, at the block level, at least *N* = 12 trials within the block had to be valid to include the block (additional *N* = 6 blocks removed). Finally, at the condition level, at least *N* = 6 trials had to include valid data to include the condition (0 conditions removed). *N* = 7 children with TH heard an alternation of dog- “tog” instead of rice- “wice”; these trials were additionally removed. On the basis of all these criteria, we removed four children with CIs from the analysis entirely. The remaining *N* = 37 children with CIs were matched to a subset of TH controls (selected semi-randomly from the approximately *N* = 160 children with TH who completed the tasks). *N* = 649 correct and mispronunciation trials remained from the *N* = 33 children with CIs (*N* = 376 trials) and *N* = 24 children with TH (*N* = 273 trials).

### Speech-language measures

To assess correlations between children’s vocabulary size, articulation, and mispronunciation sensitivity, we additionally had the children complete standardized tests of vocabulary and articulation. Vocabulary was assessed with the Expressive Vocabulary Test, 2nd edition (EVT-2) (Williams, 2007) and consonant articulation skill was assessed with the Sounds-in-Words portion of the Goldman-Fristoe Test of Articulation, 2nd edition (GFTA-2) (Goldman & Fristoe, 2000). These speech-language measures are included to explain individual differences between children within each hearing group (i.e., not to compare children with CIs and TH). Consequently, we include data from *all* children who completed the vocabulary and consonant articulation tasks (*N* = 33/37 children with CIs completed the tasks, and *N* = 24/37 children with TH completed the tasks) and not just the children who were matched for gender, maternal education, etc. (The articulation task was not measured for the children with TH at age 5.) See Table 3 for descriptive statistics of assessment results by hearing status and Supporting Information S1 for demographic information for these children.

For the articulation test, children were asked to repeat *N* = 53 picture-prompted words. Children’s productions were audio-recorded for offline scoring. *N* = 37 singleton consonants in word-initial, -medial, and -final positions (onset and coda), and *N* = 16 consonant clusters in word-initial position, were then scored. Only omissions, substitutions, and nonresponses, but not epenthesized segments, were marked incorrect. For the vocabulary test, children were presented with an image and asked to name it or provide a synonym. Our statistical modeling includes growth scale values for vocabulary (transformations of raw scores that grow linearly with age) and standard scores for articulation skills (scores normalized for sex and age; Table 3).

## RESULTS

We evaluated the mispronunciation sensitivity of children with CIs in comparison to their hearing age- and vocabulary size-matched peers. The outcome variable is the proportion of looks at the familiar object versus the unfamiliar object as a function of time (300–1800 ms after target word onset). We modeled these looking proportions using Generalized Additive Mixed Models (GAMMs). This approach has become an important tool to model time series data, such as eyetracking trajectories, because it can estimate flexible, nonlinear relationships (“smooths”) between variables such as time and relevant covariates (i.e., effects of group and/or condition; van Rij et al., 2016; Zahner et al., 2019). GAMMs are composed of (fixed) *parametric* terms that model static relationships between two variables, as is common in generalized linear modeling, and *smooth* terms that model nonlinear effects by using penalized basis functions (i.e., smoothing splines). Parametric terms can typically be interpreted from model summaries, as in traditional regression, but smooth terms must be interpreted visually. Wieling (2018) and Sóskuthy (2021) provide tutorials for GAMMs in linguistics, and Wood (2017) provides a comprehensive textbook treatment of the approach.

Generalized Additive Mixed Models (GAMMs) also allow for autocorrelation between observations to be factored into the modeling. Incorporating autocorrelation is of particular importance to eyetracking data where we anticipate large amounts of within-trial correlation between measurements over time: the area where the child is looking at 500 ms is highly correlated with where they are looking at 550 ms. As such, GAMMs are a significant improvement upon other polynomial regression models common in time series analysis such as Growth Curve Models (GCMs). The standard approach for estimating GCMs cannot factor in this inherent correlational structure within the data and, as a result, recent work has shown that they result in inflated Type I error rates (Huang & Snedeker, 2020).

The current data were analyzed in the RStudio computing environment (R version 4.0.2; R Core Team, 2020). All computing and statistical analyses are included in the GitHub repository affiliated with this project (github.com/megseekosh/ci-mispron). Visualizations were made using the ggplot2 (Wickham, 2016) and cow-plot (Wilke, 2020) packages. Modeling was conducted and presented using the mgcv (Wood, 2017), itsadug (van Rij et al., 2020), and tidymv packages (Coretta, 2022; see project documentation for package versions). For all modeling, the proportion of children’s looks to the familiar object versus the unfamiliar object was calculated for each frame (every 50 ms) and transformed to empirical logit (elog), or the log-odds of looking at the familiar object at each sample (Barr, 2008). Random effects (“factor smooths” in GAMMs) included by-participant, by-observation (first [at age 3], second [at age 4], or third [at age 5] visit to the lab), and by-item trajectories. These factor smooths modeled variability stemming from individual children and lexical items and took into account the repeated observations from some children at two different ages. To ensure assumptions were met and to avoid overfitting, model criticism was conducted using the gam.check() function; when necessary, the number of basis functions (*k* or knots) was increased.

As is common in eyetracking data, the response data were distributed with heavy tails. Consequently, all models were fit using a scaled-*t* model using the scat() link function, which substantially improved data distribution (Wood et al., 2016). Finally, for each model, autocorrelation between model residuals was calculated; all models showed high amounts of autocorrelation. These dependencies were factored into the modeling by allowing AR(1) an autoregressive error parameter that modeled the degree of autocorrelation (*rho*) between time points in each trial. Subsequent model inspection demonstrated that specifying this autocorrelation value in the model sufficiently factored out autocorrelation between residuals (Wieling, 2018).

### Evaluating the effect of phonetic detail on mispronunciation sensitivity

To evaluate how access to fine, phonetic detail may affect mispronunciation sensitivity, a series of GAMMs were fit comparing children with CIs and their hearing age- and vocabulary size-matched TH peers. *Condition* (Correct Pronunciation vs. Mispronunciation) was contrast-coded to facilitate model interpretation and the 2 × 2 relationship between *Group* (Children with CIs vs. TH) and *Condition* was modeled using ordered factors. A model with parametric and smooth terms for *Group* and *Condition* improved upon a *Condition*-only model, suggesting that children with CIs and TH responded differently to correct pronunciations versus mispronunciations.

To statistically evaluate the source of the *Group* effect (i.e., stemming from overall vs. time-varying response to the stimuli), another model was fit that included parametric terms for *Group*, and the ordered factors of *Correct Pronunciation* for children with CIs and *Correct Pronunciation* for children with TH (Wieling, 2018). These parametric effects modeled the constant effect of the covariates upon the response variable; smooth terms are centered around 0, and these parametric effects adjust these curves to center at some average proportion of looks. Smooth model terms included nonlinear effects of *Time* and *Time* by *Group*. The latter allowed us to model the nonlinear difference between the two different groups’ responses to mispronunciations. Finally, the model included difference smooths, which allowed us to separately model how each hearing group responded to correct- versus mis-pronunciations over time. See Table 4 for model summary.

**FIGURE 2:**
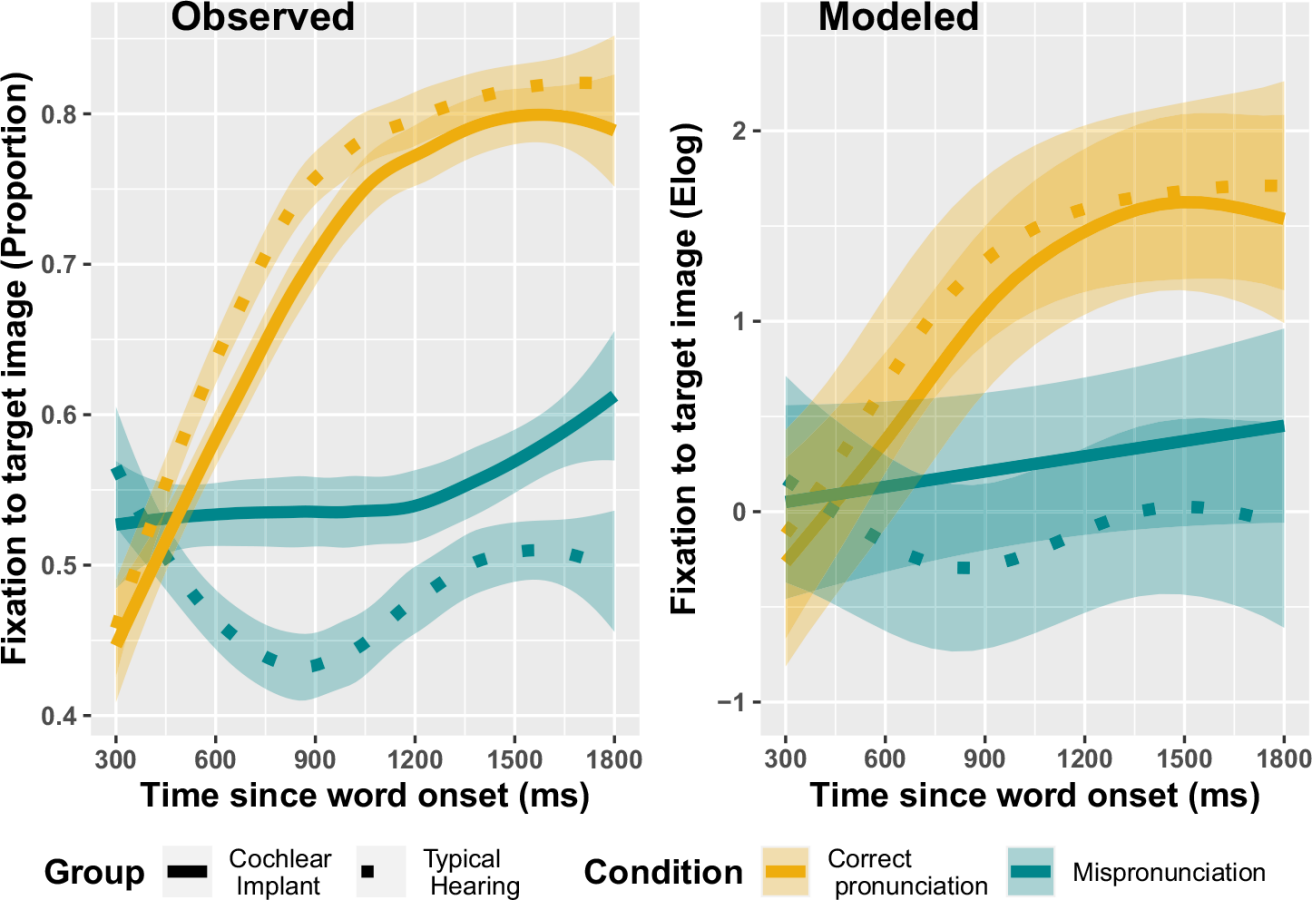
Generalized Additive Mixed Model predictions for the proportion of looks to a familiar object, by word condition and hearing status. Fixations on the y-axis are plotted as the empirical logit values (elog). Shaded ribbons represent 95% confidence intervals.

In the first part of the results, we ask: are both children with CIs and their TH matches sensitive to mispronunciations? Parametric effects in the model summary show that there are, overall, significantly more looks to the familiar photo for *Correct Pronunciation* trials than *Mispronunciation* trials, for both children with CIs and TH (CI logit Est. = 0.74, *p* < .001, proportion Est: 0.22; TH logit Est. = 1.3, *p* < .001, proportion Est: 0.33). We interpret the smooth terms by first considering effective degrees of freedom (EDF) and the significance test for each smooth. The EDF indicates how much wiggliness there is in a smooth where EDF = 1 indicates a linear relationship and a larger value indicates more wiggliness in the smooth. Interpretation of the nonlinear smooths shows that there are significant, nonlinear differences in looks to the familiar object between correct- and mis-pronunciations for children with CIs (smooth of *Time* by the ordered *Cochlear Implant; Correct*) and children with TH (smooth of *Time* by the ordered *Typical Hearing; Correct*; Figure 2). Thus, both children with CIs and TH are sensitive to mispronunciations.

**FIGURE 3:**
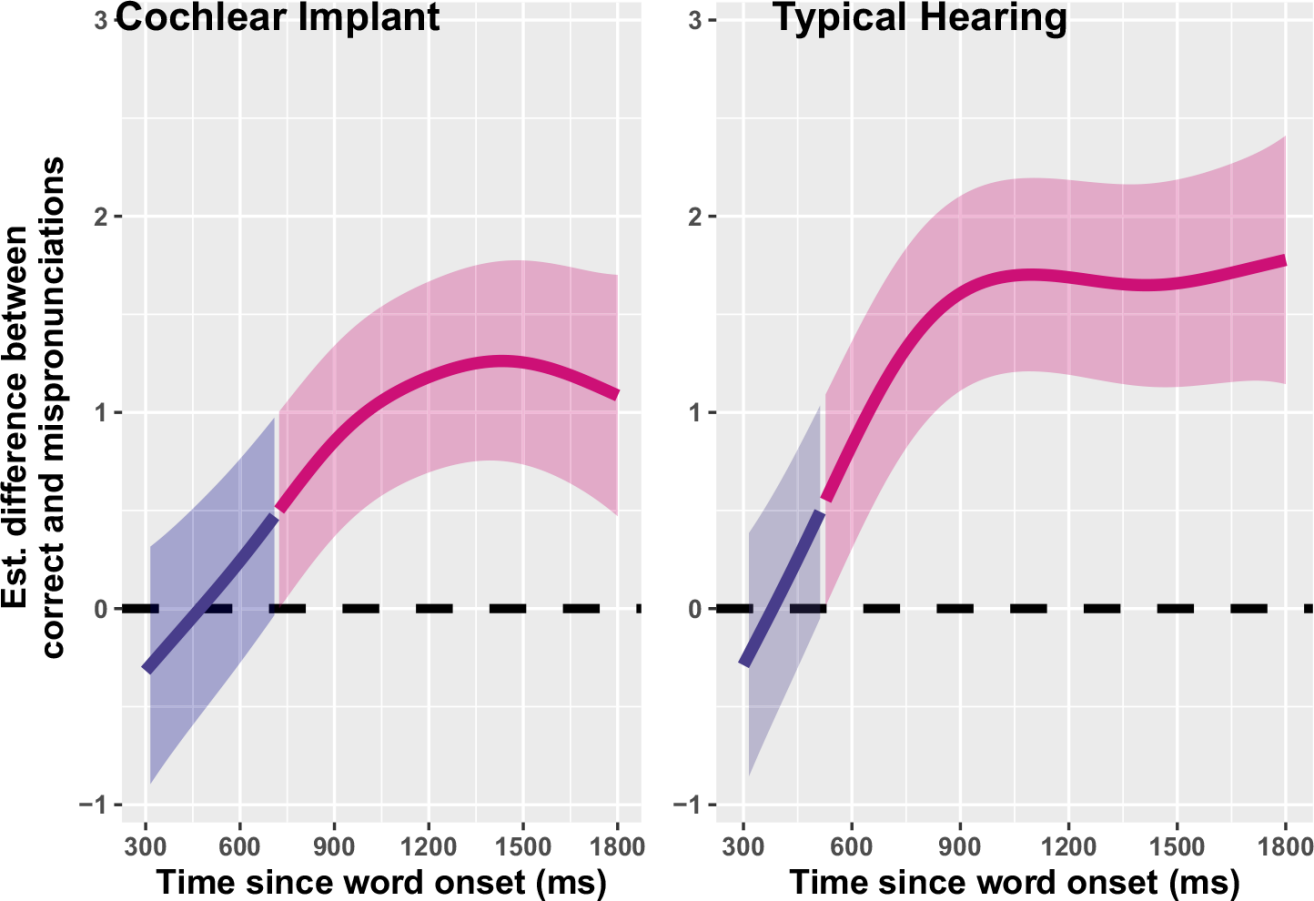
Difference smooths (Generalized Additive Mixed Model predictions) by condition (correct-vs. mis-pronunciations) for children with CIs (L) and TH (R). Pink smooths represent the point when correct- and mispronunciation smooths differ (i.e., the reliable effect of condition) for each group. Shaded ribbons represent 95% confidence intervals. A higher difference value indicates greater discrepancies between correct- and mispronunciations or greater mispronunciation sensitivity: there is a larger difference between correct- (see also difference between yellow lines in Figure 2) and mispronunciation responses (see also the difference between turquoise lines in Figure 2) for children with TH than CIs. CI, cochlear implant; TH, typical hearing.

**FIGURE 4:**
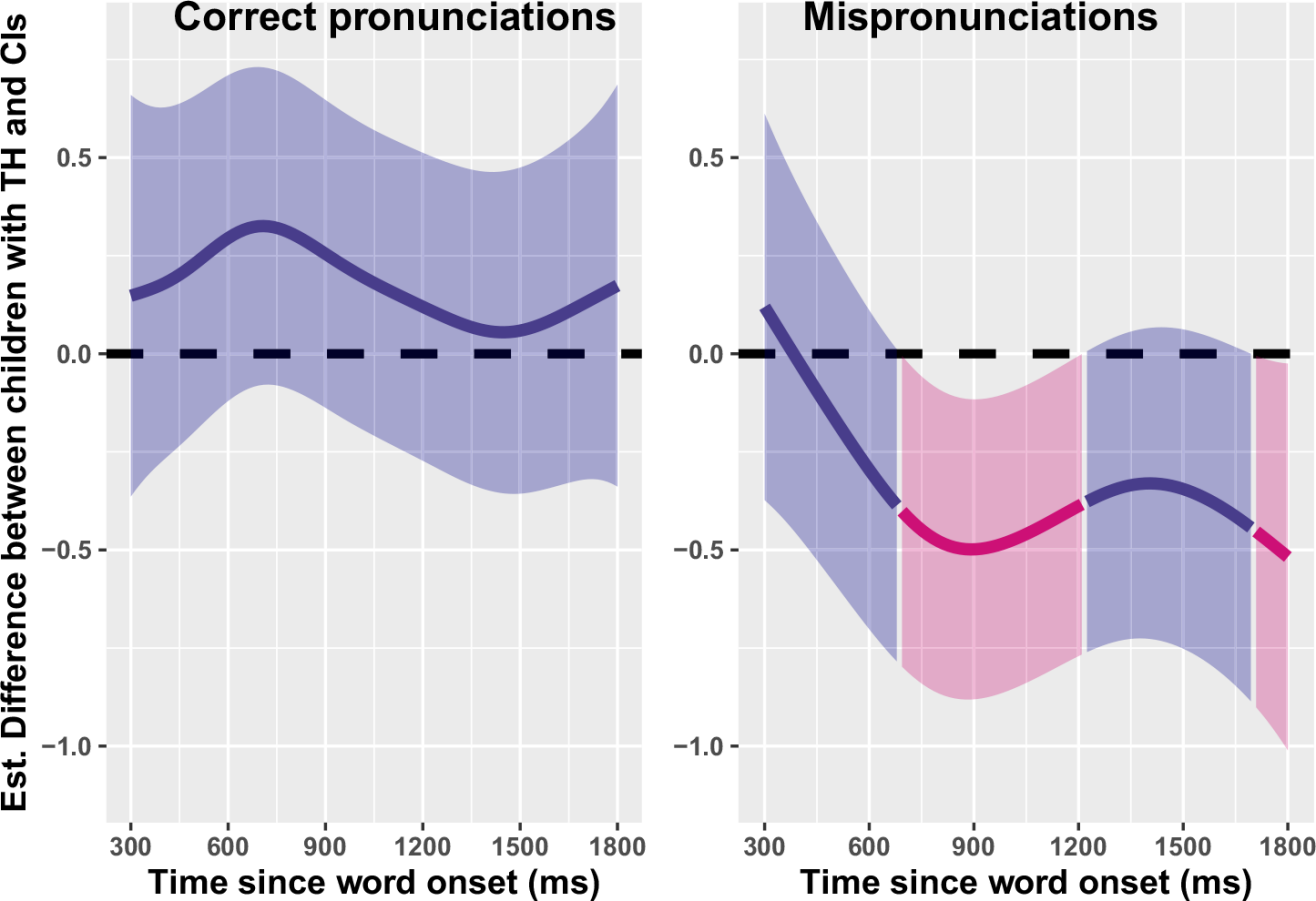
Difference smooths (Generalized Additive Mixed Model predictions) by hearing status for correct pronunciations (L) and mispronunciations (R). Pink smooths represent the point when the smoothness for children with CIs differs from children with TH (i.e., the reliable effect of the group). Shaded ribbons represent 95% confidence intervals. A higher difference value indicates larger differences between children with TH and CIs: there is an effect of the group upon mispronunciations, but not correct pronunciations. CI, cochlear implant; TH, typical hearing.

Nevertheless, the above modeling cannot tell us if these children with CIs are *less* sensitive to mispronunciations than their TH peers; the modeling demonstrates only that both groups show sensitivity. To evaluate differences in mispronunciation sensitivity by group, another GAMM was fit, with a binary difference smooth, which allowed us to evaluate the *difference* between smooths (Correct- vs. Mis-pronunciations) for children with CIs and TH, over time. Model fit included parametric effects of *Group*, as well as smooths of *Time*, *Time* by *Group*, *Time* by *Condition*, and *Time* by the ordered variable of *Group* by *Condition* (to model the difference between real- and mispronunciations for each group). Model results are plotted in Figure 3; the model summary is included in Supporting Information S1. Overall, the model-estimated difference smooths show smaller differences between correct- and mispronunciations for the children with CIs—and that these differences take longer to manifest during online processing (left panel of Figure 3). Further inspection of the first model, as plotted in Figure 3, demonstrates why this is the case. The children with CIs and TH do not respond significantly differently to correct pronunciations: once vocabulary size and hearing age are controlled, both groups of children respond similarly to correctly pronounced words. Instead, children with CIs—who are listening with a degraded speech signal via electric hearing—are less sensitive to *mis*pronunciations (Figure 4), resulting in smaller difference smooths between correct- and mis-pronunciations.

### Explaining individual differences in mispronunciation sensitivity

Having established that children with CIs are less sensitive to mispronunciations than their TH peers, we next correlated the children’s responses with two different standardized speech-language assessments: expressive vocabulary size (EVT-2) and spoken phonetic/articulatory accuracy (GFTA-2). Because we took an individual differences approach, we examined the children with CIs and TH separately.

We modeled the effects of vocabulary size and articulation on the children who completed both assessments (*N* = 33 with CIs and *N* = 24 with TH) by using stepwise GAMM fitting. Specifically, we assessed the nonlinear interaction between *Time*, *Condition*, and *Vocabulary Score*/*Phonetic Accuracy* to evaluate if children’s vocabulary sizes and/or phonetic accuracy predicted their looks to the target over time for the correct- and mis-pronunciation conditions. As before, all models included factor (random) smooths by participants, observation (visit to the lab), and item. Each additionally included a difference smooth of *Time* and *Participant* by *Condition* (*Correct-* vs. *Mispronunciation*). A baseline model was fit with a parametric term for *Condition* (estimating the average looking probability in each condition), smooth terms for *Time* and *Time* by *Condition*, as well as a nonlinear interaction (tensor product) of *Time* and *Child Age* by *Condition*. In all models, we included the Age by Condition tensor product smooth term because our child-level variables (vocabulary score and phonetic accuracy) are confounded with age, and we wanted to evaluate the potential influence of these speech-language abilities independent of child age. We modeled Chronological Age for the children with TH. Since the age of implantation and years of device use are strong predictors of speech-language outcomes among children with CIs, we additionally modeled Age at Implantation and Hearing Age for the children with CIs, but Chronological Age resulted in the best model fit.

We fit the three-way smooth interaction of *Time*, *Condition*, and *Vocabulary Score* and *Time*, *Condition*, and *Phonetic Accuracy* using tensor product terms. For the children with TH, neither the vocabulary nor phonetic accuracy terms improved upon a baseline model controlling for the child’s age. This result indicates that, for the children with TH, mispronunciation sensitivity—the difference in looks to the target image in correct- versus mispronunciation conditions—is not moderated by vocabulary size or phonetic accuracy over and above age effects. For the children with CIs, the best model fit included *Phonetic Accuracy*; *Vocabulary Score* did not improve upon model fit. The final model summary for the children with CIs is included in Supporting Information S1.

**FIGURE 5:**
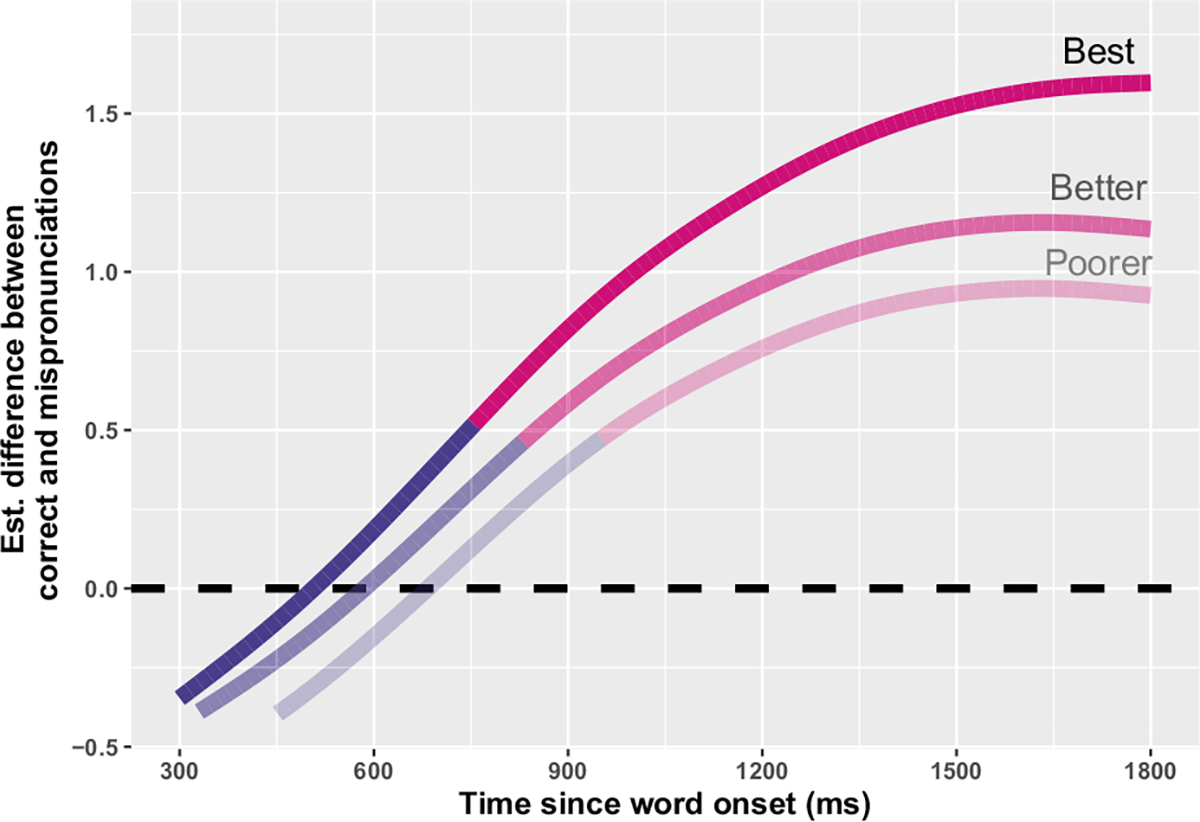
Difference smooths (Generalized Additive Mixed Model predictions) between correct- and mis-pronunciations for children with cochlear implants, by standardized articulation score. Pink smooths represent the point when correct- and mis-pronunciations smooths significantly differ (i.e., reliable effect of condition). Children were divided into tertiles by score, with smooths representing the median score for children with poorer (median score = 57), better (72), and best (96) articulation scores.

**FIGURE 6:**
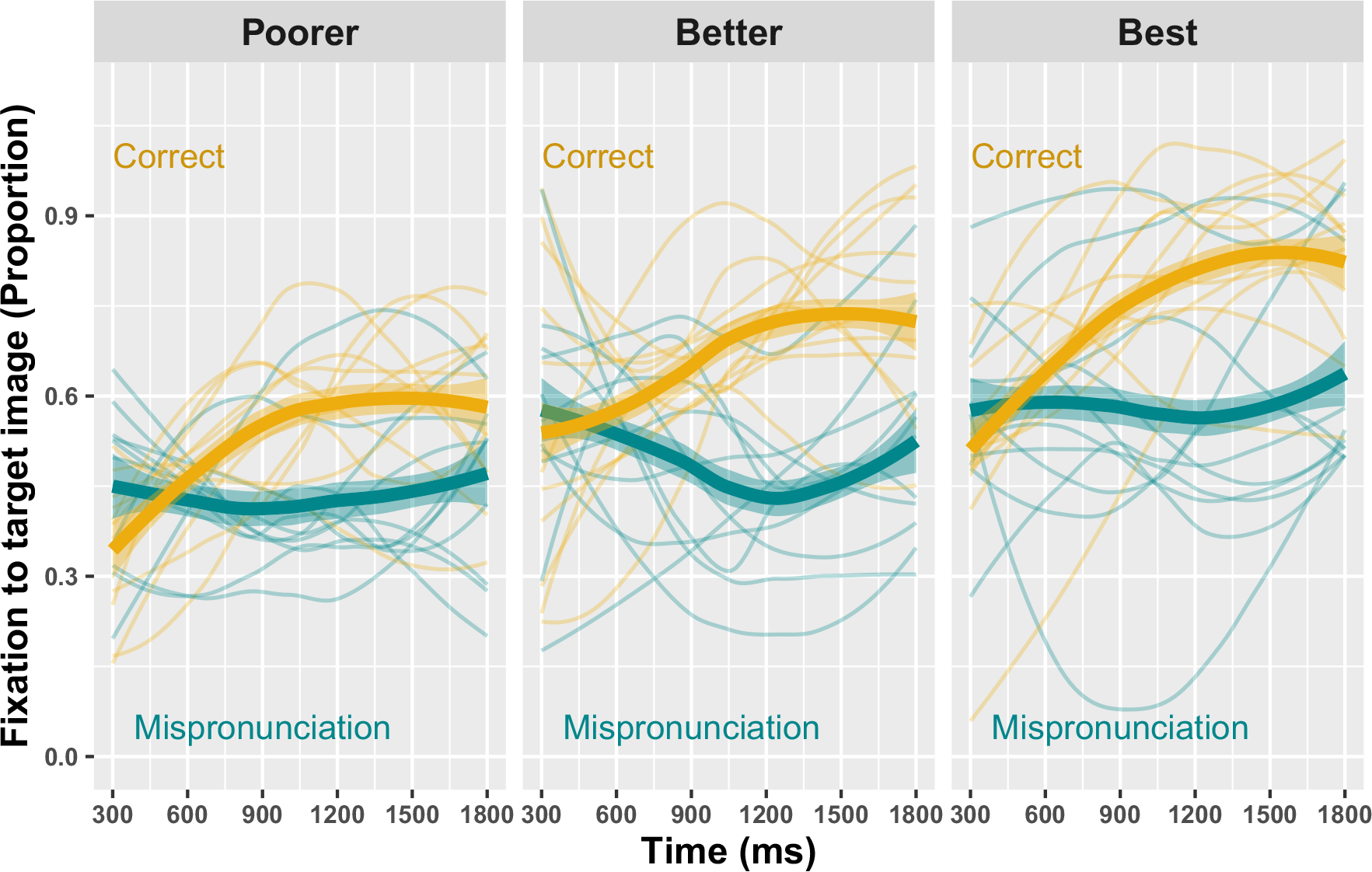
Raw response trajectories for the proportion of looks to familiar objects for children with cochlear implants, by word condition and standardized articulation score. Children were divided into tertiles by score: poorer (median score = 57), better (72), and best (96) articulation scores.

Given the multiple nonlinear effects at play, it is necessary to plot the model predictions in order to interpret GAMM outputs, in particular how phonetic accuracy mediates mispronunciation sensitivity for children with CIs. To facilitate the interpretation of the nonlinear three-way interaction, the children with CIs were divided into tertiles by vocabulary score and phonetic accuracy. Predictions from the model, by articulatory tertile, are plotted in Figure 5 and raw response curves are plotted in Figure 6. The model predictions demonstrate that children with better articulation scores show larger differences between looks to the target for correct- versus mispronunciations (higher overall *y*-intercept value) and that these children show significant differences between correct- and mis-pronunciations slightly earlier in the analysis window (cross-over from purple to pink smooth occurs sooner in the analysis window). Thus, for the children with CIs, phonetic/articulatory accuracy predicts mispronunciation sensitivity, independent of age and language ability.

## DISCUSSION

This study asked how preschoolers learn to process variation in speech by taking advantage of the unique sensory experiences of CI users. Our objective was to see how much detail children with CIs were sensitive to during online speech-processing tasks. Are fine-grained representations required to process variable speech? The unique sensory profile of CI users allows us to examine this question from a new angle. The electric hearing generated by the CI results in a degraded speech signal which allowed us to assess how (lack of) regular access to fine, phonetic detail affected preschoolers’ speech processing while controlling for lexical knowledge (vocabulary size).

We carried out a variant of the mispronunciation sensitivity paradigm where children responded to correct pronunciations (*soup*) and mispronunciations (“shoup”). Our analysis resulted in two main findings. First, we found that when matched for lexical knowledge (vocabulary size) and lifetime oral language exposure (years of hearing experience), children with CIs and TH processed correctly pronounced words along a similar time course. Differences between hearing groups instead stemmed from responses to mispronunciations: children with TH tended to look equally at the familiar and unfamiliar objects (equivocating), or they looked more to the unfamiliar image (treating it as a novel word). In contrast, children with CIs preferred the familiar image. Thus, they showed reduced sensitivity to mispronunciations and were more likely to disregard them. Second, for the children with CIs, sensitivity to mispronunciations was correlated with phonetic skill (articulatory accuracy on a standardized assessment), but not vocabulary size: children with higher articulation scores showed greater sensitivity to mispronunciations, in line with other work that has established perception-production links in children of this age (Rvachew, 1994; Shiller et al., 2010).

Taken together, these results suggest that *all* children—those with and without CIs—use their lexical knowledge to process correct pronunciations. However, children rely on fine phonetic detail to process speech variation. In the absence of access to a rich, reliable phonetic signal, such as that generated from acoustic hearing, children do not develop the same sensitivity to speech variation. We elaborate upon these points below.

### Articulatory skill, not vocabulary, predicts sensitivity

Contrary to our hypothesis, we did not find a reliable relationship between vocabulary size and mispronunciation sensitivity for either children with CIs or TH. This finding runs counter to previous work that has documented such a relationship in 2- to 3-year-olds (Law & Edwards, 2015; Swingley, 2016). In that work, vocabulary is cited as one possible mechanism that children may use to develop sensitivity to speech variation: children with larger vocabularies are thought to have more well-specified phonological representations due, in part, to the demands that denser phonological neighborhoods place upon representations (Edwards et al., 2004; Sosa & Stoel-Gammon, 2012; Stoel-Gammon, 2011). This effect extends in the other direction as well as children with more advanced speech (notable during the early babbling periods) go on to develop larger expressive vocabularies and language (Rescorla & Ratner, 1996; Vihman, 2014).

Yet, the modeling here did not demonstrate a relationship between children’s vocabulary size and mispronunciation sensitivity—a relationship was only found for phonetic accuracy and then only for children with CIs. For both children with CIs and TH, there was certainly sufficient variability between children to capture a potential effect of vocabulary (growth scale value score range 42–159 for all of the N = 33 children with CIs for all of the N = 24 children with TH, although all children with TH had above-average vocabulary sizes for their age). Consequently, differences between the current study and previous work could stem from the age group tested. Children in the current study are several years older than those previously studied, meaning that facilitative effects of vocabulary may only manifest within a certain developmental window. In further support of this idea are longitudinal data showing a facilitative effect of expressive vocabulary size for mispronunciation sensitivity at three years of age, but not four or five (Mahr, 2018). Thus, our null result of vocabulary is not at odds with previous work and is instead further evidence that vocabulary only predicts sensitivity to speech variation for a certain period in early development, before age 4.

Modeling did demonstrate a correlation between phonetic accuracy and mispronunciation sensitivity for children with CIs. The children with higher articulation scores looked more quickly and reliably to the target word when they heard a correct pronunciation, acting quickly, and decisively. This effect manifests visually in Figure 4 with the advantage of correct pronunciations over mispronunciations increasing with articulation ability. We interpret this finding as suggesting that the children who are skilled at capturing the phonetic signal during online processing—and are sensitive to disruptions in it—are the same children who are skilled at articulating sounds during speech production. At the age group studied (34–66 months), children who perform poorly on standardized tests of articulatory ability are no longer doing so *purely* for motoric reasons (i.e., inability to front the tongue dorsum). Instead, we believe that poor performance on both tasks (mispronunciation sensitivity and phonetic accuracy) indicates that a child with (a) CI(s) is less practiced at interpreting the electric hearing signal and manipulating it into a phonological representation that they can use in speech processing and production. Alternatively, or perhaps in addition, children who are better at articulation could have greater mispronunciation sensitivity because they are more adept at perceiving speech production targets in their environments. In any case, the electric signal that these children hear certainly allows them to learn and process words—after all, the children with CIs (as a group) processed correct pronunciations along a similar timescale as their vocabulary-matched peers with TH. Nevertheless, the fact that the standardized assessment of phonetic accuracy can explain variation in mispronunciation sensitivity suggests a single developmental mechanism underlying the children’s ability to produce and perceive individual phonemes. The result suggests that well-specified phonological representations drive accuracy in speech production and sensitivity in speech processing.

### Sensitivity to speech variation matters for word learning

To learn words and phonemes, children must learn appropriate amounts of sensitivity to the speech used in their environments. As we have outlined in this paper, children must have access to a detailed (acoustic) speech signal to process variation. What happens when a degraded speech signal results in underspecified phonological representations? What are the consequences of learning to process speech variation, and thus learn new words? Havy et al. (2013) posed a similar question and asked 3- to 6-year-olds with CIs to make novel word mappings from two phonetically similar words (e.g., /suk/ and /ʃuk/). The children with CIs performed better when the words were more distinct (3-feature difference vs. 1-feature difference) and their performance correlated with years of CI use, a pattern that the authors attribute to the children’s “challenges of recruiting fine phonetic sensitivities when forming a new referential word object link” (p. 188) which corresponds to the notion of phonological insensitivity explored in this work.

We demonstrated here that vocabulary cannot help compensate for underspecified representations—at least after a certain stage in development. Even controlling for vocabulary size, children who classify novel phonological neighbors (“shoup”) as variants of a word (*soup*) will struggle to map novel words to referents in their environments. These children might not consider variants like “shoup” to be completely homophonous with *soup*. The children with CIs studied here were, after all, sensitive to mispronunciations. Indeed, it is remarkable, given the degree of signal degradation, just how closely the children with CIs approximated the patterns of children with TH. Instead, what these results say is that children with CIs have mildly reduced sensitivity to mispronunciations even several years post-implantation.

From a clinical perspective, this result means that young children with CIs may need additional support to learn similar-sounding words. Perhaps, they would require more exposure to a close phonological neighbor to map it to a referent in their environment, especially when those words differ in coda position as this is where children with CIs have especially weak phonological awareness skills (Nittrouer et al., 2016). Or perhaps these children would need to hear repeated exemplars of this close phonological neighbor, spoken by multiple interlocutors around them, in order to disentangle a potential variant of a known word from a new word to be mapped.

In either case, the child with CIs would take longer to learn new words, especially those that fall into dense phonological neighborhoods. Unfortunately, the child’s lexicon is replete with dense phonological neighborhoods (cf. Charles-Luce & Luce, 1990). In typical development, children learn dense phonological neighborhoods *first* (Carlson et al., 2014; Jones & Brandt, 2019; Storkel, 2004), especially in production. So, for children systematically exposed to a degraded signal, one consequence could be the developmental trajectory of phonological neighborhood restructuring (Storkel, 2002). Charles-Luce and Luce (1990) originally postulated that children had sparser phonological neighborhoods than adults due to the under-specification of their phonological representations. In the decades since, research on sensitivity to mispronunciation among children with TH has shown that children *do* have relatively well-specified phonological representations (Swingley & Aslin, 2002; White & Morgan, 2008) and *do* learn dense neighborhoods (Storkel, 2004). However, for children with electric hearing, the current results suggest a developmental path more akin to that originally outlined in Charles-Luce and Luce (1990): electric hearing results in the kind of underspecified representations once proposed in Charles-Luce and Luce (1990) and thus children with CIs may have sparser neighborhoods than even their vocabulary-size matched peers with TH.

### A note about the developmental trajectory of mispronunciation sensitivity

In the mispronunciation sensitivity paradigm, the most mature response to hearing a mispronunciation (“shoup”) is to look at the opposing image (i.e., to look *away* from an image of soup). This response indicates that a child noticed the /s/ > [ʃ] substitution and that the substitution disrupted their lexical access of *soup*.

However, as mentioned repeatedly in this work, sometimes efficient speech processing requires *ignoring* variation. Mature listeners and interlocutors regularly factor out speech variation stemming from differences in vocal tract morphology, speaking rate, and geographical dialect—the inability to do so would completely hinder communication. Now, a substitution such as /s/ > [ʃ] does, clearly, cross a phonemic threshold that phonetic variants on a word do not. For example, the centroid frequency of /s/ lowers in rounded, back-vowel contexts, such as *soup*, and yet this variation does not disrupt word recognition. In fact, adult listeners know to compensate for the lowered /s/ in these environments (Mann & Repp, 1980). Still, while the *most* developmentally immature response to one phonological feature substitution (“shoup”) is to continue looking at the image of soup, suggesting that the child has not noticed the mispronunciation, and a *more* mature response is to recognize the mispronunciation and look away from the image of soup, the *most* mature response would be to (1) initially recognize the mispronunciation (look away from the soup) and then (2) recover from it (look back at the soup). This processing pattern would indicate that the child has recognized the mispronunciation—so they have relatively well-defined phonological representations—but has had sufficient experience processing speech to know to disregard some word variants. We call this a “reject and reconsider response” to speech variation, a mature processing strategy where children would revise their original hypothesis concerning word identity. Indeed, Mahr (2018) found this mature strategy in typically developing 5-year-olds when they examined a subset of mispronunciation trials where the child initially fixated on the familiar image. Nevertheless, the degree and time course of the revision should vary by a number of factors including the dialect spoken, the speaker source, and the rate of speech.

We were interested in exploring a potential revise response in our own data. It seemed unlikely that such a pattern would emerge in the children with CIs who only have, on average, 33 months of hearing experience. So, we instead explored the pattern in children with TH. These analyses were purely exploratory, not confirmatory, and future work should extend our analysis in a hypothesis-driven manner.

**FIGURE 7:**
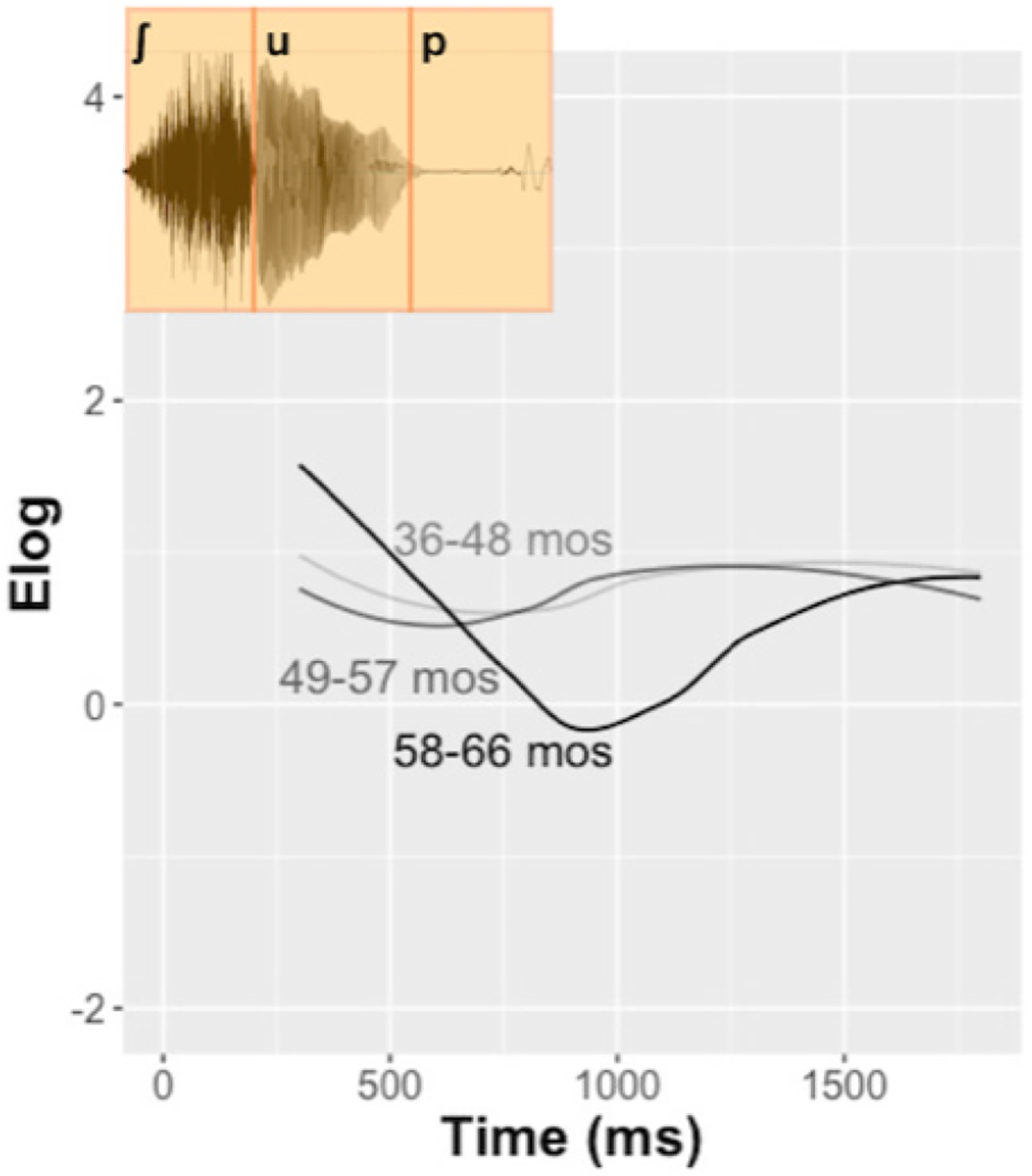
Raw response trajectories and audio stimulus for the proportion of looks to *soup* upon hearing the mispronunciation “shoup” for children with typical hearing. Children were divided into tertiles by chronological age.

We again divided the children with TH into tertiles, in this instance by chronological age. Figure 7 plots raw response curves to the mispronunciation “shoup” with the time course of the audio stimulus. We plot this for the *soup*–“shoup” mispronunciation in particular because this substitution is phonetically grounded and observable in running speech: the centroid frequencies of fricatives such as /s/ are known to lower (more closely approximating [ʃ]) before back, round vowels. Our exploratory analysis shows that older children (58–66 months) exhibit more of the reject and reconsider response than either group of younger children, indicating that they revised their original lexical hypothesis. Younger children (36–48 and 49–57 months) have a flatter response with a slight increase in looking at the target image (soup) over time.

These results are exploratory, but they do suggest that not only does overall sensitivity to mispronunciations increase with age (Von Holzen & Bergmann, 2021), but the processing strategy changes with age and cognitive maturity as well. Since a wealth of research has, by now, demonstrated that infants and children *are* sensitive to vowel and consonant mispronunciations, in various phonological environments, we now encourage future work examining the time course and processing strategies underlying this sensitivity.

### Limitations

This study is limited in a number of ways. First, outcomes among children with CIs are highly variable, owing to differences in age-at-implantation, regularity of device use, degree of residual hearing, and other factors. Although at *N* = 33 preschoolers between 36–66 months (a relatively small age range for a study on CIs), this study had more explanatory power than many others on young children with CIs, our field is in great need of large-scale (> N = 100 children) studies to properly model all of the different aspects that predict developmental outcomes in this population. Our sample likewise included 4 children with CIs who were sampled twice, 1 year apart. And while our statistical modeling accounted for these repeated measures, the smaller number of *unique* children studied may limit the generalizability of these results to other children, both with and without CIs.

## CONCLUSION

Variation in spoken language is rampant. To learn the sounds and words used in the language spoken around them, young children must learn to contend with this variation. This study asked if children must have access to fine phonetic detail to process speech variation by examining how children who receive CIs—who hear via a degraded, electric speech signal—process variable speech. Our results showed that 3- to 5-year-old children of all hearing backgrounds could rely on their lexical knowledge to process known words (soup). However, even after carefully matching the children with CIs to children with TH by vocabulary size and years of oral language exposure, the children with CIs were less sensitive to variable pronunciations (“shoup”). This result suggests that the degraded CI signal impacts speech processing and that children are not able to overcome these challenges via vocabulary growth or hearing experience. The children with CIs’ ability to process variable pronunciations was additionally correlated with their spoken phonetic accuracy, suggesting a single developmental mechanism underlying the ability to produce *and* process individual phonemes. Thus, while preschoolers can rely on their lexical knowledge to process known words, they must have access to a robust speech signal, and well-specific phonological representations, to process variable speech.

## Supplementary Material

I

II

## Figures and Tables

**TABLE 1:** Participant demographic information (N = 19 matches).

Hearing status	Girls, boys	Chronological age: months	Hearing age: months	Maternal education level	EVT-2 GSVs	EVT-2 standard score

Children with CIs	13, 6	56.3 (6.7) 44–66	44.7 (8.7) 29–56	6.26 (0.56)	134.84 (12) 112–159	102.63 (13.37) 84–131
Children with TH	13, 6	Not applicable	45.5 (7.5) 36–57	6.05 (0.62)	132.68 (10.88) 117–150	114.58 (10.38) 98–134

Abbreviations: CI, cochlear implant; EVT-2, Expressive Vocabulary Test, 2nd edition; GSVs, growth scale values; TH, typical hearing.

To facilitate matching, *N* = 4 children with CIs and *N* = 5 children TH were observed at two time points (e.g., at ages 3 and 4). Mean (SD), range.

**TABLE 2:** Lexical stimuli and manipulations used in eyetracking paradigm.

Contrast and manipulation	Target word	Mispronunciation transcription	Novel word

/s/ > [ʃ]	Soup	[ʃup]	Cheem [ʧim]
/ʃ/ > [s]	Shoes	[suz]	Geev [giv]
/g/ > [d]	Girl	[dɝl]	Shan [ʃæn]
/d/ > [g]	Duck	[gʌk]	Neydge [neıʤ]
/k/ > [g]	Cake	[gek ]	Pum [pʌm]
/r/ > [w]	Rice	[waıs]	Bape [bep]

**TABLE 3:** Summary statistics of standardized speech-language measures for all children, by hearing status (*N* = 33 children with CIs and *N* = 24 with TH).

Hearing status	EVT-2 standard score	EVT-2 GSVs	GFTA-2 standard score

Children with CIs	95.7 (18.71) 46–127	120.76 (25.99) 42–159	73.61 (19.33) 39–107
Children with TH	116.17 (12) 88–134	140.46 (15.46) 117–164	90.04 (12.04) 67–113

Mean (SD), range. Scores are used to assess individual differences within groups and as such, all children are included (including those who were not matched); children are not matched by hearing experience, gender, socioeconomic status, or language.

Abbreviations: CI, cochlear implant; EVT-2, Expressive Vocabulary Test, 2nd edition; GSVs, growth scale values; TH, typical hearing.

**TABLE 4:** Model summary predicting the difference between the proportion of looks to the familiar object by word condition and hearing status.

Parametric coefficients	Estimate	SE	*t*-Value	*p*-Value

Intercept (cochlear implant: mispronunciation)	0.28	0.19	1.44	.15
Typical hearing	−0.36	0.20	−1.82	.07
Typical hearing: correct pronunciation	1.30	0.23	5.59	<.001
Cochlear implant: correct pronunciation	0.74	0.23	3.21	.001

Smooth terms	Effective degrees of freedom	Ref. df	*F*-value	*p*-value

s(Time)	0.00	0.00	0.59	.99
s(Time,Cochlear Implant)	1.00	1.00	2.46	.12
s(Time,Typical Hearing)	3.91	4.90	1.72	.13
s(Time,Typical Hearing: Correct)	6.11	7.29	8.08	<.001
s(Time,Cochlear Implant: Correct)	4.27	5.40	6.44	<.001
s(Time,Child)	65.93	340.00	0.43	<.001
s(Time,Item)	34.74	106.00	0.68	<.001
s(Time,Observation)	0.00	26.00	0.01	<.001

## Data Availability

The analyses presented here were not preregistered. All data and analytic code to replicate these analyses are publicly accessible at github.com/megseekosh/ci-mispron. The materials (stimuli) necessary to attempt to replicate the findings presented here are available from the first author.

## APPENDIX

**TABLE A1:** Audiological information from the *N* = 25 unique children with cochlear implants studied.

Participant	Matched to child with TH?	Chronological age	Age at hearing loss	Age at activation	Hearing age	Etiology	Device formation	Activation order

300E	Y	57	0	13	44	Genetic	Bilateral	Simultaneous
302E	Y	37	0	13	24	Unknown	Bilateral	R-L
303E	Y	65	6	13	52	Unknown	Bilateral	Simultaneous
304E	Y	48	0	12	36	Genetic	Bilateral	R-L
305E	Y	44	0	22	22	Unknown	Bilateral	R-L
306E	Y	49	0	8	41	Unknown	Bilateral	R-L
307E	Y	44	0	15	29	Genetic	Bilateral	R-L
309E	Y	59	0.5	7	52	Genetic	Bilateral	Simultaneous
311E	Y	62	9	13	49	Unknown	Bilateral	L-R
314E	Y	38	10	17	21	Unknown	Bilateral	R-L
608 L	Y	55	0.5	9	46	Connexin 26	Bilateral	Simultaneous
665 L	Y	40	0	12	28	Genetic	Bilateral	R-L
801E	Y	39	1.5	15	24	Unknown	Bilateral	Simultaneous
804E	Y	56	0	7	49	Genetic	Bilateral	Simultaneous
809E	Y	64	6	8	56	Meningitis	Bilateral	R-L
301E	N	53	0	45	8	Unknown	Bilateral	R-L
308E	N	37	0	13	24	Genetic	Bilateral	Simultaneous
310E	N	52	Unknown	23	29	Genetic	Bilateral	Simultaneous
312E	N	57	0	24	33	Genetic	Bilateral	R-L
679 L	N	58	0	29	29	Genetic	Bimodal	n/a
800E	N	65	30	37	28	Genetic	Bilateral	Simultaneous
803E	N	41	0	34	7	Unknown	Bimodal	n/a
806E	N	42	14	34	8	Genetic	Unilateral	L
807E	N	51	10	22	29	Mondini malformation	Bimodal	n/a
808E	N	37	0	6	31	Genetic	Bilateral	Simultaneous

## Abbreviations:

CI: cochlear implant
EDF: effective degrees of freedom
EVT-2: Expressive Vocabulary Test, 2nd edition
GAMMs: Generalized Additive Mixed Models
GCMs: Growth Curve Models
GFTA-2: Goldman-Fristoe Test of Articulation, 2nd edition
TH: typical hearing
