# Supplementary material for: Preschoolers rely on rich speech representations to process variable speech
Source: Child Dev. Author manuscript; Available in PMC 2023 Jul 10. (PMC10330571; doi:10.1111/cdev.13922)
Supplement: I [file NIHMS1907732-supplement-I.pdf]

Supplementary Materials I for “Preschoolers rely on rich speech representations to process variable speech”

Photo norming

In a pretest conducted in their preschool classroom, N=30 children, all typically-developing per teacher report, correctly identified the familiar items. Children were presented with 4 different items and were asked to name each one and point to the photo of the target familiar item (e.g. *soup*). All of the familiar items were correctly or appropriately labeled (i.e., “food” instead of *soup*) by at least 80% of the children. As in Law and Edwards (2015), N=14 of these children additionally completed an open-set test to identify the unfamiliar items.

Demographic information

Table 1

Demographic information of children who completed standardized speech-language measures, by hearing status (N=33 children with CIs and N=24 with TH). Mean (SD), range.

Group	Girls, Boys	Chronological Age: months	Hearing Age: months	Maternal Education Level
Children with CIs	21, 12	50.91 (10.01) 34–66	33.33 (14.91) 5–56	5.76 (1.09)
Children with TH	15, 9	52.71 (10.81) 36–66	52.71 (10.81) 36–66	5.67 (1.34)

**Parametric coefficients**	**Estimate**	**Std. Error**	**t-value**	**p-value**
Intercept (Cochlear Implant: Mispronunciation)	0.28	0.19	1.44	0.15
Typical Hearing	-0.37	0.20	-1.82	0.07
**Smooth terms**	**edf**	**Ref. df**	**F-value**	**p-value**
s(Time)	0.00	0.00	6.26	< 0.001
s(Time,Cochlear Implant)	1.00	1.00	0.14	0.71
s(Time,Typical Hearing)	3.86	4.84	3.02	0.01
s(Time,Cochlear Implant Correct - Cochlear Implant Mispronunciation)	5.68	6.71	6.71	< 0.001
s(Time,Typical Hearing Difference - Cochlear Implant Difference)	5.86	7.05	2.19	0.04
s(Time,Child)	66.37	341.00	0.42	< 0.001
s(Time,Item)	34.61	107.00	0.72	< 0.001
s(Time,Observation)	0.00	27.00	0.01	< 0.001

Table 2

Predicting the degree of mispronunciation sensitivity by hearing status.

**Parametric coefficients**	**Estimate**	**Std. Error**	**t-value**	**p-value**
Intercept (Mispronunciation)	0.08	0.17	0.47	0.64
Correct	0.74	0.19	3.87	0.001
**Smooth terms**	**edf**	**Ref. df**	**F-value**	**p-value**
s(Time)	0.00	9.00	0.00	0.50
s(Time,Correct)	3.23	9.00	2.45	< 0.001
s(Age)	0.58	9.00	0.15	0.01
te(Time,Age; Correct)	3.27	24.00	0.42	0.003
te(Time,Phonetic Accuracy; Correct)	3.18	24.00	0.45	0.001
s(Time,Child)	48.16	296.00	0.28	< 0.001
s(Time,Item)	10.78	106.00	0.24	< 0.001
s(Time,Observation)	0.49	26.00	0.03	0.02
s(Time,Child; Correct)	0.00	296.00	0.00	< 0.001

Table 3

Predicting the degree of mispronunciation sensitivity by phonetic accuracy score.

References

Law, F., & Edwards, J. R. (2015). Effects of Vocabulary Size on Online Lexical Processing by Preschoolers. *Language Learning and Development*, *11*(4), 331–355. doi: 10.1080/15475441.2014.961066
