# Supplementary material for: Preschoolers rely on rich speech representations to process variable speech
Source: Child Dev. Author manuscript; Available in PMC 2023 Jul 10. (PMC10330571; doi:10.1111/cdev.13922)
Supplement: II [file NIHMS1907732-supplement-II.pdf]

Supplementary Materials II for "Preschoolers rely on rich speech representations to process variable speech"

Results

Analysis plan

This supplememntary material reports a comparison between how children with cochlear implants (CIs) and TH (typical hearing) respond to real words and novel words in the eyetracking paradigm. As in the results section of the paper, the comparison groups were matched for hearing age, gender, maternal education, and vocabulary size. The outcome variable is the proportion of looks to the familiar object versus the unfamiliar object, over time (300–1,800 ms after target word onset), which we modeled using Generalized Additive Mixed Models (GAMMs). See the Results section in the paper for a description of how to interpret GAMMs, how these GAMMs were fit, and all details on the computing environment that generated these results.

Comparing children's responses to real words and novel words

A series of GAMMs were fit comparing children with CIs and their hearing age- and vocabulary size-matched TH peers. **Condition** (Real Word vs. Novel Word) was contrast-coded to facilitate model interpretation and the 2x2 relationship of **Group** (Children with CIs vs. TH) and **Condition** was modeled using ordered factors. A model with parametric and smooth terms for **Group** and **Condition** improved upon a **Condition**-only model, suggesting that children with CIs and TH responded differently to real- versus novel words.

To statistically evaluate the source of the **Group** effect (i.e., stemming from overall vs. time-varying response to the stimuli), another model was fit that included parametric terms for **Group**, and the ordered factors of *Real Word* for children with CIs and *Real Word* for children with TH (Wieling, 2018). These parametric effects modeled the constant effect (the intercept) of the covariates upon the response variable. Smooth model terms included non-linear effects of **Time** and **Time** by **Group**. The latter allowed us to model the non-linear difference between the two different groups' responses to novel words. Finally, the model included difference smooths, which allowed us to separately model how each hearing group responded to real versus novel words, over time. See Table 1 for model summary.

**Parametric coefficients**	**Estimate**	**Std. Error**	**t-value**	**p-value**
Intercept (Cochlear Implant: Novel word)	-0.89	0.16	-5.55	< 0.001
Typical Hearing	-0.38	0.14	-2.78	0.01
Typical Hearing: Real word	2.47	0.23	10.80	< 0.001
Cochlear Implant: Real word	1.91	0.23	8.42	< 0.001
**Smooth terms**	**edf**	**Ref.df**	**F-value**	**p-value**
s(Time)	2.23	2.21	0.50	0.66
s(Time,Cochlear Implant)	1.00	1.00	0.00	.99
s(Time,Typical Hearing)	2.50	3.22	0.83	0.50
s(Time,Typical Hearing; Real word)	5.98	7.29	16.74	< 0.001
s(Time,Cochlear Implant; Real word)	4.42	5.66	16.81	< 0.001
s(Time,Child)	0.00	340.00	0.00	< 0.001
s(Time,Item)	22.07	106.00	0.48	< 0.001
s(Time,Observation)	1.74	26.00	0.07	0.25

Table 1

Model summary predicting the difference between proportion of looks to the familiar object by word condition and hearing status.

Parametric effects in the model summary show that there are, overall, significantly more looks to the familiar photo for *Real Word* trials than *Novel Word* trials, for both children with CIs and TH (CI logit Est.=1.91, p<.001, or proportion Est: 0.48; TH logit Est.=2.47, p<.001, or proportion Est: 0.62). We interpret the smooth terms by first considering effective degrees of freedom (EDF) and the significance test for each smooth. The EDF indicates how much wiggliness there is in a smooth where EDF=1 indicates a linear relationship and a larger value indicates more wiggliness in the smooth. Interpretation of the non-linear smooths shows that there are significant, non-linear differences in looks to the familiar object between real and novel words for children with CIs (smooth of **Time** by the ordered **Cochlear Implant; Real word**) and

children with TH (smooth of **Time** by the ordered **Typical Hearing; Real Word**) (Figure 1).

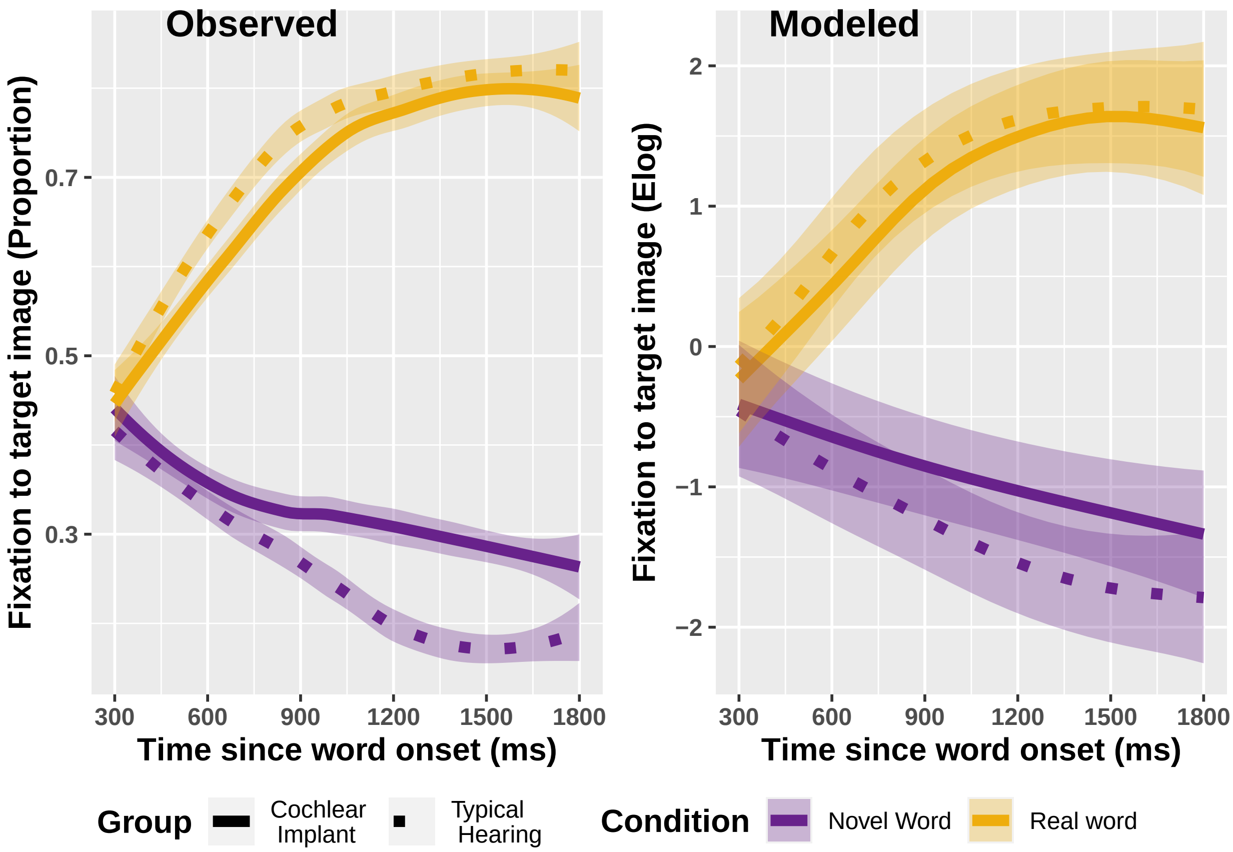

Figure 1. Observed data and GAMM predictions for proportion of looks to familiar object, by word condition and hearing status. Fixations on the y-axis are plotted as the empirical logit values (elog). Shaded ribbons represent 95% confidence intervals.

To evaluate differences in word learning (response to novel word items) by group, another GAMM was fit, with a binary difference smooth, which allowed us to evaluate the *difference* between smooths (Real- vs. Novel Words) for children with CIs and TH, over time. Model fit included parametric effects of **Group**, as well as smooths of **Time**, **Time** by **Group**, **Time** by **Condition**, and **Time** by the ordered variable of **Group** by **Condition** (to model the difference between real and novel words for each group). Model results are plotted in Figure 2; see Table 2 for the model summary. Overall, the model-estimated difference smooths show smaller differences between real and novel words for the children with CIs—and that these differences take slightly longer to manifest during online processing (left panel of Figure 2). Further inspection of the first

model, as plotted in Figure 3, demonstrates why this is the case. The children with CIs and TH do not respond significantly differently to real words (which we already demonstrated in the primary analyses in the paper): once vocabulary size and hearing age are controlled, both groups of children respond similarly to real words. Instead, children with CIs look less to the novel item in response to novel words (right panel of Figure 3), resulting in smaller difference smooths between real and novel words.

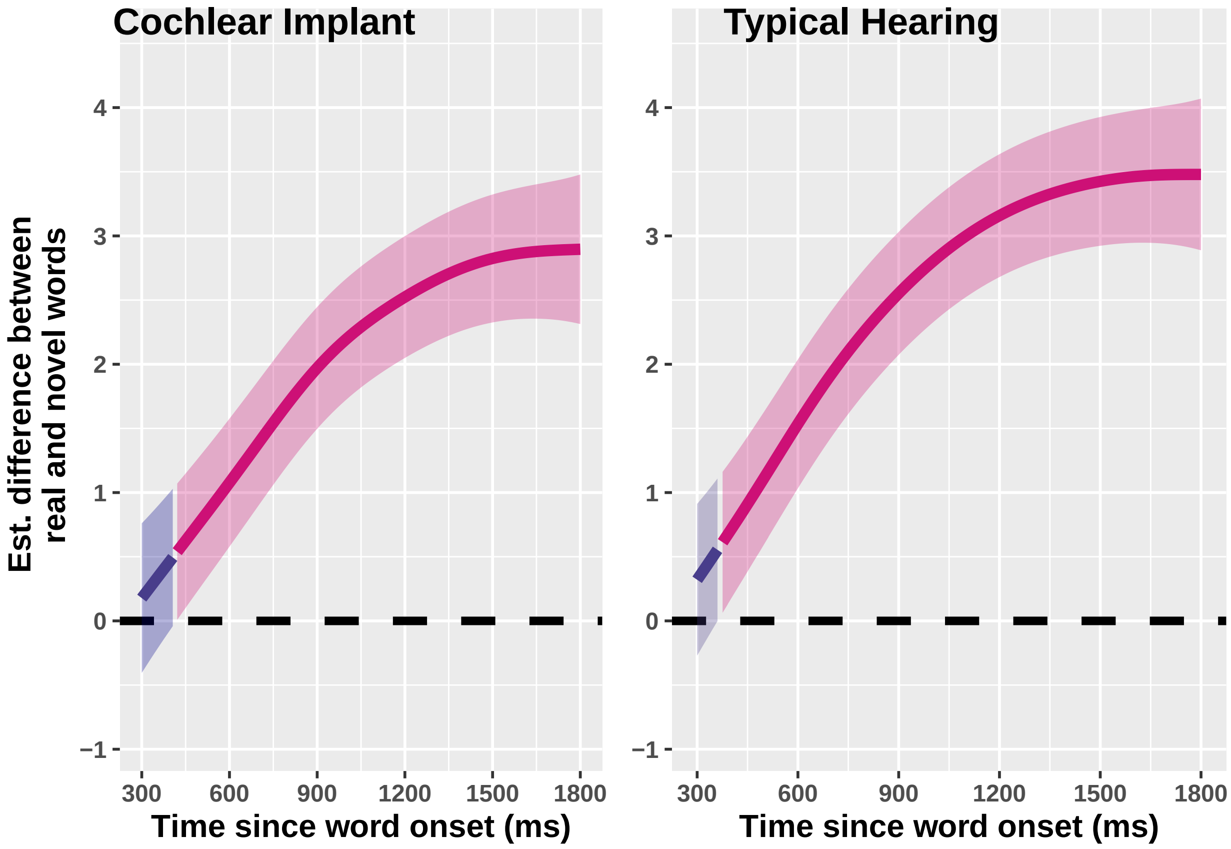

Figure 2. Difference smooths (GAMM predictions) by condition (real- vs. novel words) for children with CIs (L) and TH (R). Pink smooths represent the point when real and novel smooths differ (i.e., reliable effect of condition) for each group: there is a larger difference between real and novel word responses for children with TH than CIs. Shaded ribbons represent 95% confidence intervals.

Explaining individual differences in response to novel words

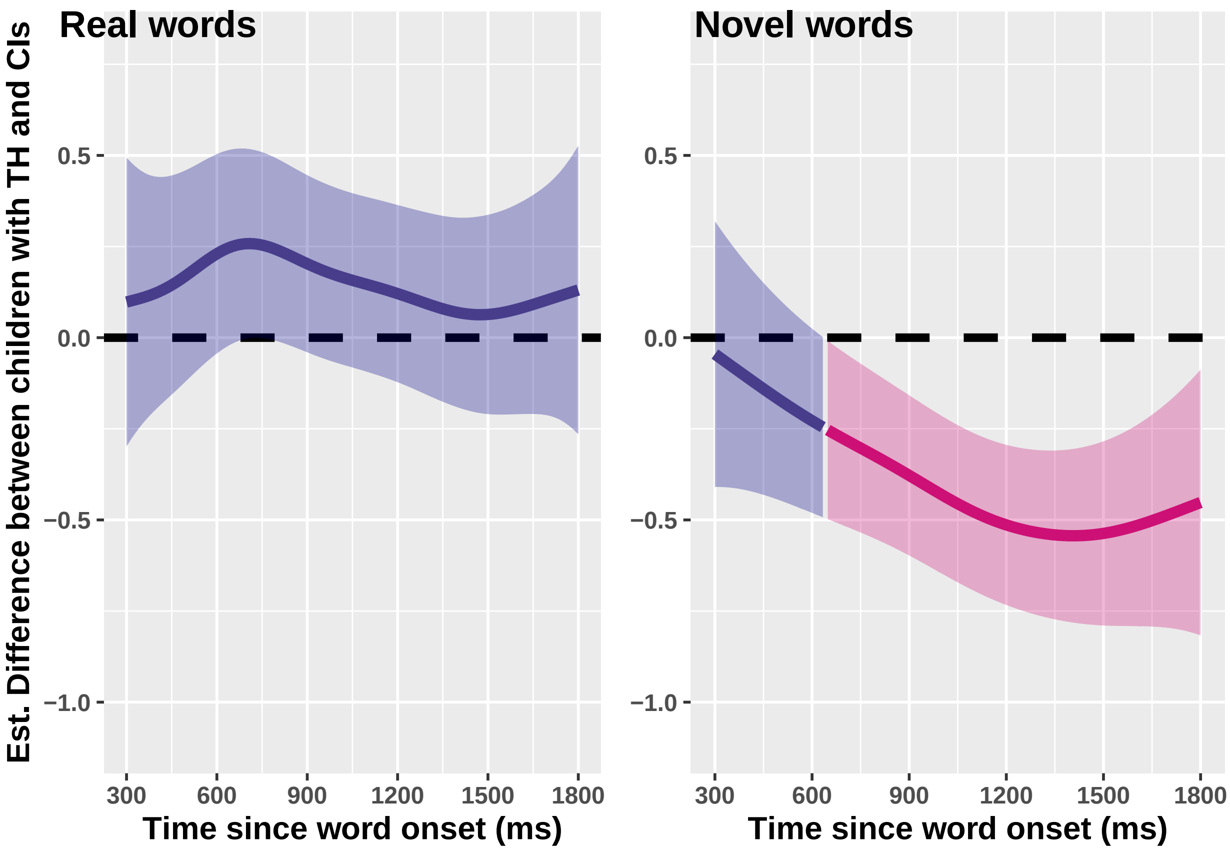

Figure 3. Difference smooths (GAMM predictions) by hearing status for real and novel words. The pink smooth represents the point when the smooth for children with TH differs from children with CIs (i.e., reliable effect of group): there is an effect of group upon response to novel words (R plot: see also difference between purple lines in Figure 1), but not real words (L plot: see also difference between yellow lines in Figure 1). Shaded ribbons represent 95% confidence intervals.

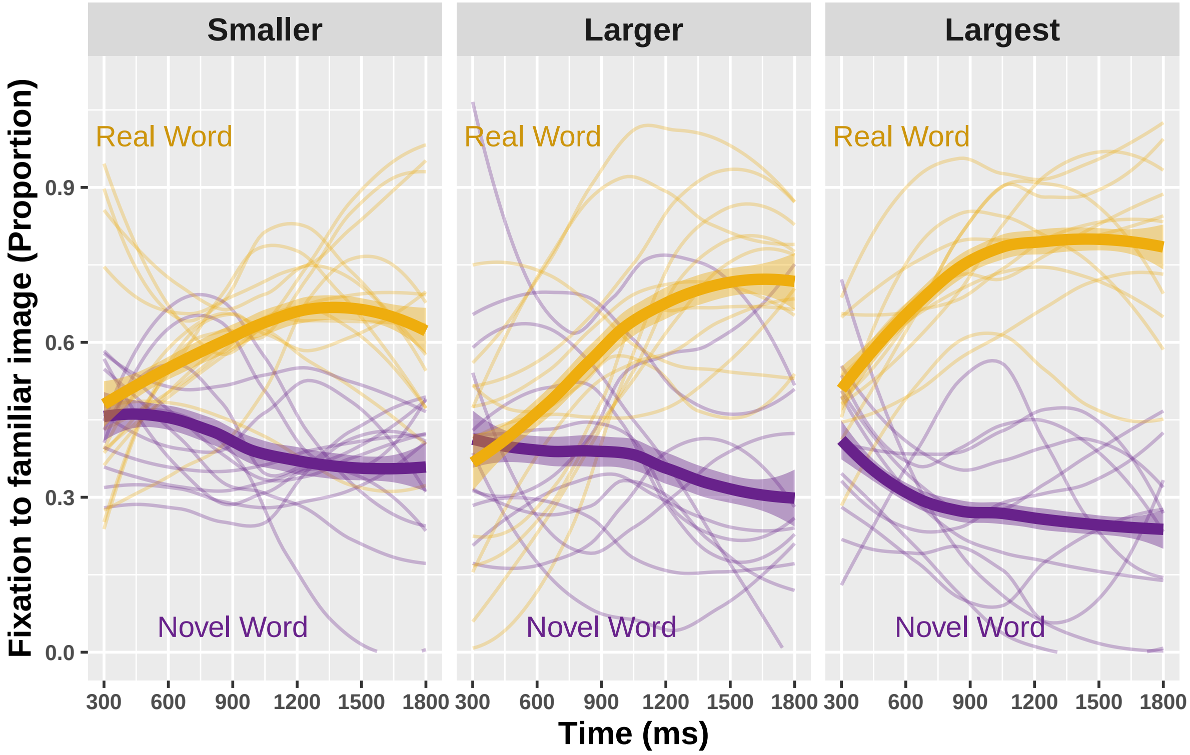

Figure 4. Raw response trajectories for proportion of looks to familiar object for children with CIs, by word condition and standardized vocabulary score. Children were divided into tertiles by score: smaller (median score=106), larger (129), and largest (143) vocabulary scores.

Parametric coefficients	Estimate	Std. Error	t-value	p-value
Intercept (Cochlear Implant: Novel Word)	-0.91	0.16	-5.69	< 0.001
Typical Hearing	-0.34	0.13	-2.56	0.01
**Smooth terms**	**edf**	**Ref.df**	**F-value**	**p-value**
s(Time)	1.7049	2.42	4.06	0.01
s(Time,Cochlear Implant)	1.00	1.00	23.85	< 0.001
s(Time,Typical Hearing)	1.51	1.87	17.08	< 0.001
s(Time,Cochlear Implant Real - Cochlear Implant Novel Word)	6.68	7.92	23.31	< 0.001
s(Time,Typical Hearing Difference - Cochlear Implant Difference)	2.00	2.00	3.86	0.02
s(Time,Child)	0.01	340.00	0.00	0.54
s(Time,Item)	21.91	107.00	0.51	< 0.001
s(Time,Observation)	0.94	26.00	0.05	0.10

Table 2

Predicting response difference between real and novel words, by hearing status.

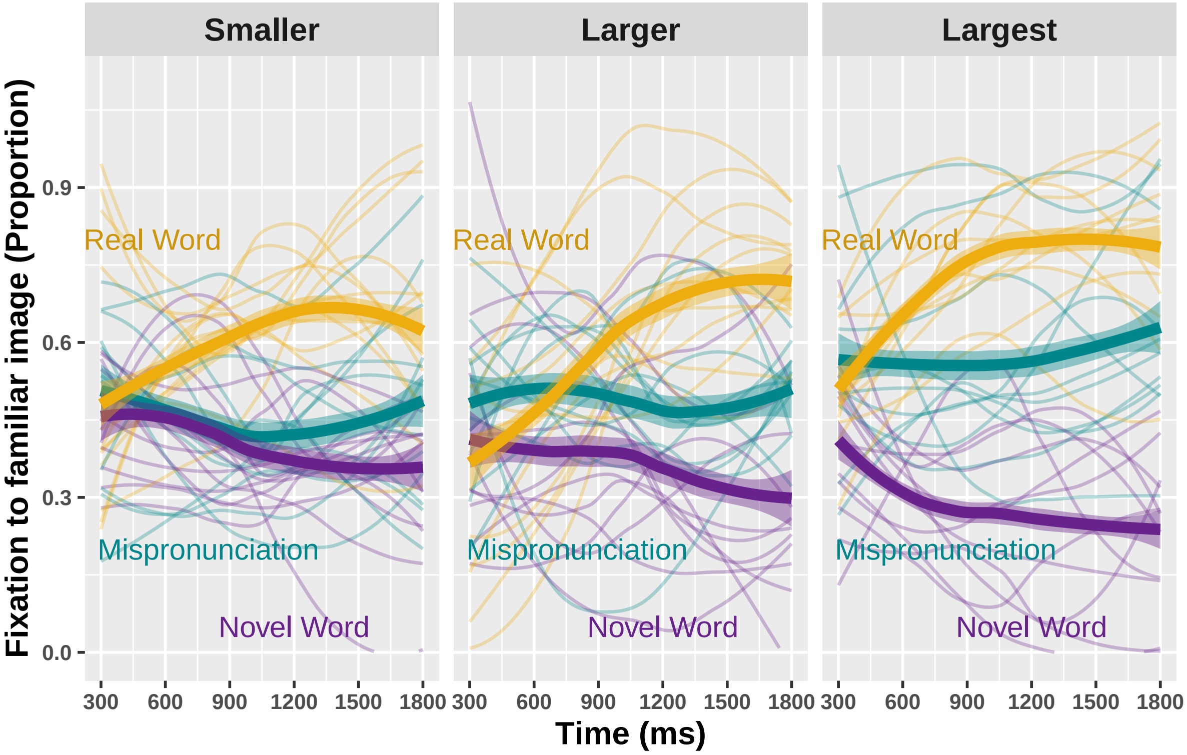

Figure 5. Raw response trajectories for proportion of looks to familiar object for children with CIs, by all three word conditions (correctly-pronounced real word, mispronounced word, novel word) and standardized vocabulary score. Children were divided into tertiles by score: smaller (median score=106), larger (129), and largest (143) vocabulary scores.

References

Wieling, M. (2018). Analyzing dynamic phonetic data using generalized additive mixed modeling: A tutorial focusing on articulatory differences between L1 and L2 speakers of English. *Journal of Phonetics*, *70*, 86–116. doi: 10.1016/j.wocn.2018.03.002
